# Do the Reasons People Drink Alcohol Aid Our Understanding of Sociodemographic Differences in Alcohol‐Free and Low‐Alcohol Consumption? A Path Analysis on a Cross‐Sectional Study of Adult Alcohol Drinkers in Great Britain

**DOI:** 10.1111/dar.70159

**Published:** 2026-05-05

**Authors:** Lucy Burke, Colin Angus, Jamie Brown, Inge Kersbergen

**Affiliations:** ^1^ University of Sheffield Sheffield UK; ^2^ University College London London UK; ^3^ University of Bath Bath UK

**Keywords:** alcohol drinking, drinking behaviour, health inequalities, public health

## Abstract

**Introduction:**

In the UK, consumption of alcohol‐free (< 0.05% ABV) and low‐alcohol (≤ 1.2% ABV; NoLo) drinks is more prevalent among heavier drinkers and socially advantaged groups. If heavier drinkers are substituting alcoholic drinks with NoLo drinks, this could improve public health. However, socioeconomic differences in consumption could exacerbate alcohol‐related health inequalities. Socioeconomic groups vary in their reasons for drinking alcohol, with less advantaged individuals more likely to drink alcohol to cope. This study examined whether alcohol drinking motives can help explain differences in NoLo consumption.

**Methods:**

A total of 2549 adults residing in Great Britain provided data on at least monthly NoLo consumption, hazardous drinking (AUDIT‐C), alcohol drinking motives, social grade, education, age and gender, via the Alcohol Toolkit Study. Path analysis explored mediating effects of drinking motives between sociodemographic characteristics, hazardous drinking and NoLo consumption.

**Results:**

Drinking alcohol to conform, education and hazardous drinking were positively associated with NoLo consumption. Drinking alcohol to cope with depression was a serial mediator between social grade and NoLo. Drinking to cope with depression, more frequently reported among lower social grades, weakened the positive relationship between hazardous drinking and NoLo consumption (*β* = −0.001, 95% CI −0.002, −0.000). Enhancement and social motives also weakened this relationship, partially mediating pathways between age, gender, education and NoLo consumption.

**Discussion and Conclusions:**

While hazardous drinking is positively associated with NoLo consumption, for those drinking to cope with depression, for enhancement or for social reasons, this effect diminishes, potentially limiting the public health potential for those who drink for these reasons, including disadvantaged groups.

## Key Point Summary

- There is increasing evidence that alcohol‐free and low‐alcohol (NoLo) drinks are more likely to be consumed by the more socially advantaged, which could lead to an increase in health inequalities
- This study seeks to understand whether the reasons people drink alcohol, captured using items from the Drinking Motives Questionnaire—Revised, can help explain sociodemographic differences in NoLo consumption, with a particular interest in understanding the socioeconomic differences observed.
- Drinking alcohol to cope with depression was found to mediate the relationship between social grade and NoLo consumption by weakening the positive relationship between hazardous drinking and NoLo consumption.
- Drinking alcohol for enhancement and for social reasons also reduced the relationship between hazardous drinking and NoLo consumption.
- Alcohol drinking motives may help us better understand who is likely to substitute alcohol with NoLo and benefit from public health policies to increase the availability of NoLo drinks to reduce alcohol‐related harm.

## 1 Introduction

Reducing alcohol‐related harm is a critical public health priority. In 2023, 10,473 people died in the UK from alcohol‐specific causes, the highest number on record, and an increase of 38% since before the COVID‐19 pandemic [1]. Internationally, alcohol harms are disproportionately experienced by the poorest groups, explaining 4%–5% of the inequality gap in life expectancy between the least and most socially advantaged [2, 3, 4], therefore, it is imperative that public health policies aim to reduce health inequalities in tandem with improving overall population health.

Consecutive UK governments have proposed working with industry to increase the availability of alcohol‐free and low‐alcohol (NoLo) drinks as a public health strategy [5, 6]. NoLo drinks are defined as alcoholic or alcoholic‐type (e.g., beer, wine, spirits) beverages that contain a lower amount of alcohol by volume (ABV) than ‘standard’ alcoholic drinks. In the UK, NoLo drinks encompass both ‘alcohol‐free’ (currently < 0.05% ABV) and ‘low‐alcohol’ (≤ 1.2% ABV) products [7]. This combined threshold is used in the current study. Internationally, the threshold for ‘No’ or ‘Lo’ varies by jurisdiction [8]. Due to these inconsistencies, we will state the ABV threshold used for papers cited that deviate from our defined threshold.

While NoLo drinks are inappropriate for those needing to completely abstain from alcohol, they do not lead to intoxication and its associated harms, making them a potentially beneficial alternative for the general drinking population [8]. The pathway to benefit is via substitution. If consumers replace standard alcoholic drinks with NoLo alternatives, this will reduce their overall alcohol consumption and consequently alcohol‐related harm, particularly if these products appeal to at‐risk groups.

An increasing number of studies suggest that heavier drinking groups are those most likely to purchase and consume NoLo products, supporting their potential as a harm reduction strategy through substitution. In Great Britain, a large representative cross‐sectional study (*n* = 7691) using self‐report data found that riskier drinkers were significantly more likely to consume NoLo drinks regularly, with at‐risk drinkers being nearly 4 times more likely than non‐drinkers to have ever consumed a NoLo drink [9]. Similar findings have been reported internationally. A US self‐report study using a convenience sample (*n* = 1906) found non‐alcoholic consumption (< 0.5% ABV) was significantly and positively associated with alcohol consumption (adjusted odds ratio 1.46, 95% confidence interval [CI] 1.17–1.83). Sixty‐eight percent of respondents who screened positive for alcohol use disorder attributed drinking less alcohol due to their non‐alcoholic beverage use [10]. In Finland, a longitudinal study (*n* = 47,066) analysing retail purchasing data from loyalty customers of a major retailer for alcohol‐free beers (< 1.2% ABV) also determined that non‐alcoholic beer purchases were most prevalent in the groups with the highest volumes of regular beer purchase [11]. Collectively, these findings suggest that if NoLo drinks are successfully used to substitute standard alcohol, they could potentially reduce alcohol‐related harms amongst heavier drinking groups.

The potential public health benefit of NoLo substitution is complicated by observed sociodemographic disparities in NoLo consumption. A consistent finding is that NoLo purchasing and consumption is positively associated with social advantage, captured through indices such as level of education, employment or financial circumstances. This socioeconomic patterning is currently more consistent than evidence on age or gender differences, where differences are equivocal [9, 10, 11, 12, 13, 14]. The studies discussed above [10, 11, 12] all found a socioeconomic gradient associated with NoLo consumption, in addition to levels of alcohol consumption. This pattern is corroborated by additional studies. A cross‐sectional representative study in Poland (*n* = 1114) found consumption of non‐alcoholic beer (< 0.5% ABV) was significantly associated with higher odds of being in employment [13]. Similarly, a UK purchasing study (*n* = 79,411 households) found purchasing of zero‐alcohol beer (< 0.05% ABV) was associated with higher social grades [14]. These findings raise concerns that promoting NoLo products for harm reduction at the expense of alternative policies could exacerbate health inequalities if there *is* a substitutive effect, because those who are less advantaged are less likely to consume them. In order to understand this potential imbalance, it is important to consider why these differences occur.

When evaluating alcohol policies, British policymakers recommend accounting for the reasons why people drink alcohol [15, 16, 17, 18]. Alcohol drinking motives have been operationalised in several questionnaires [19, 20, 21]. The most well‐validated measure is Cooper's (1994) Drinking Motives Questionnaire—Revised (DMQ‐R), developed from Cox and Klinger's alcohol motivation model [19, 22, 23, 24, 25, 26]. Four alcohol drinking motives are identified: enhancement, social, conformity, and coping. Cross‐sectional studies in the UK, using these dimensions, have found alcohol drinking motives mediate sociodemographic differences in alcohol consumption, with males being more likely to drink alcohol to conform and for social and enhancement reasons, while less socially advantaged drinkers being more likely to report drinking alcohol to cope [16, 17]. This measure may therefore help elucidate the potential impact of an alcohol policy on health inequalities.

The relevance of using drinking motives to better understand NoLo consumption is supported by an increasing body of literature. Drinking for conformity versus enhancement have emerged as key themes differentiating between NoLo uptake and avoidance. Qualitative studies from Australia and the UK have consistently found that NoLo consumers acknowledge social participation and adhering to social norms as key benefits of these beverages [27, 28, 29, 30, 31]. Amongst a sample of 16 adults in Australia who had successfully reduced their alcohol consumption, alcohol‐free drinks (< 0.5% ABV) were found to enable these ex‐drinkers to masquerade as ‘drinkers’, facilitating their reduction attempts [28]. A more recent Australian study including 5 online focus groups (*n* = 44) of adolescents found adolescents believed that NoLo drinks may help people avoid social stigma associated with abstinence [30]. Studies conducted in the UK, including a small self‐selected sample of regular consumers of NoLo drinks (*n* = 15), a larger study with 10 focus groups (*n* = 49) representing the demographic spread in England, and diverse NoLo consumption patterns, and pregnant people (*n* = 18), found NoLo drinks facilitated social occasions, enabling participation where alcohol consumption was typical, and allowing those not drinking alcohol to avoid scrutiny from peers [27, 29, 31]. These findings are consolidated by cross‐sectional survey data. A study using the Global Drug Survey, an international survey of self‐selected participants, found heavier drinkers who consumed NoLo beverages (using the UK threshold of ≤ 1.2%) used them to conceal their sobriety [32]. Similarly, Bowdring et al.'s US study found 26% of those who drank non‐alcoholic beverages (< 0.5%), reported doing so to blend in socially [10]. They may be particularly appealing to pregnant people for this reason, with 69% of NoLo (≤ 1.2% ABV) consumers in a UK survey of 2092 pregnant/recently pregnant people consuming NoLo drinks for this reason [33]. We directly observed this in our earlier work, showing that, in a representative sample from Great Britain, regular NoLo consumption was associated with endorsement of drinking alcohol to conform, after accounting for sociodemographic characteristics and alcohol consumption [34].

Studies exploring reasons for not consuming NoLo drinks found non‐consumers often did not see the point of NoLo drinks if the goal was to feel inebriated [29, 30, 32, 35], reinforcing the distinction between external and internal motives of alcohol consumption and their relationship with NoLo consumption.

Given the established sociodemographic disparities in NoLo consumption [9, 10, 11, 12, 13, 14] and the evidence linking alcohol drinking motives to NoLo consumption behaviour [27, 28, 29, 30, 32, 33, 34], drinking motives represent a plausible mechanism to help explain the observed sociodemographic patterning. To date, no studies have explicitly tested this, with a recent systematic review highlighting better understanding of NoLo consumption patterns as a research priority [36].

**FIGURE 1:**
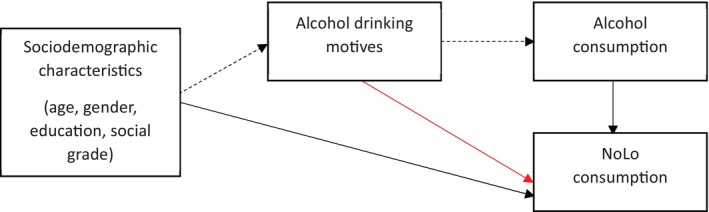
Linking pathways to alcohol and alcohol‐free and low‐alcohol (NoLo) consumption. 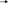: Established pathways to NoLo consumption [9]. 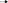: Established pathways to alcohol consumption [16, 17]. 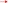: Direct association between drinking alcohol to conform and NoLo consumption [34].

The current study addresses this evidence gap by investigating the mediating role of alcohol drinking motives in explaining the association between sociodemographic characteristics and NoLo consumption amongst a representative sample of adults in Great Britain. We hypothesise that drinking motives will mediate the sociodemographic differences in NoLo consumption, following the conceptual model illustrated in Figure 1. We utilise the dimensions of Cooper's DMQ‐R [19], but distinguish between coping‐depression and coping‐anxiety motives, consistent with research on socioeconomic status and harmful drinking [37].

Our primary hypothesis was that drinking to cope with depression would mediate the relationship between social grade and NoLo consumption. We expected respondents from lower social grades would be more likely to drink to cope with depression [16, 17], and drinking NoLo would not satisfy this motive [27, 29], explaining some of the socioeconomic differences in consumption. Non‐directional hypotheses explored whether alcohol drinking motives served as mediators or serial mediators (via hazardous alcohol consumption) for age, gender, education and social grade. Our data came from the Alcohol Toolkit Study [38], a cross‐sectional, nationally representative survey of adults aged 16 and over in Great Britain, using path analysis to explore potential mediating effects.

## 2 Method

### 2.1 Design

This cross‐sectional study recruited adults aged 16 and over from the February 2023 and April 2023 waves of the Alcohol Toolkit Study [38], a monthly, nationally representative, telephone survey of adults residing in Great Britain. The sampling process for the Alcohol Toolkit Study is designed to recruit a study population that is representative of the adult population in Great Britain across key demographics, including age, gender, social grade, working status, prevalence of children in the household and region [38]. Rim (marginal) weighting adjusted the influence of each participant's response so that the sample's known characteristics matched the latest reliable population figures (e.g., UK Census data), ensuring the results were generalisable to the broader population [39].

Alongside routinely administered questions capturing respondent demographics and alcohol use, respondents also reported how often they consumed NoLo drinks. Questions capturing respondents' alcohol drinking motives (*n* = 5) were included for the selected waves.

### 2.2 Sample

A total of 4089 adults completed the survey. To be eligible for this study, respondents needed to have drunk alcohol at least once in the previous 12 months as recorded by the Alcohol Use Disorder Identification Test (AUDIT‐C, [40]), *n* = 2920 (71.4%). The following respondents were removed: those who reported they consumed NoLo drinks more frequently in particular circumstances (e.g., on/off trade) than overall (*n* = 163), those who reported ‘don't know’ for any of the alcohol drinking motives items (*n* = 189), and those who described their gender ‘in another way’, (removed due to small numbers, *n* = 13), and missing data (*n* = 6). There remained a sample of 2549 (87.3%, see Figure S1 in the Supporting Information for a participant flow‐chart). This provided reliable estimates of the pathways of interest and sufficient power to detect standardised path coefficients (*β*) greater than 0.10 (40).

### 2.3 Measures

#### 2.3.1 NoLo Drinking Behaviour

Frequency of NoLo consumption was measured as a single item [9]. Participants were asked ‘How often do you have an alcohol‐free or low‐alcohol drink (beer, wine, cider, spirits or other type of alcoholic drink under 1.2% ABV)?’. Participants responded on an 8‐point scale, ranging from Never—almost every day. Due to small numbers of responses at higher frequencies, responses were recoded as a binary variable—less than monthly/at least monthly to capture whether respondents were regular consumers of NoLo drinks or not.

#### 2.3.2 Alcohol Drinking Motives

Alcohol drinking motives were captured using five items from Cooper et al.'s DMQ‐R [19]. This measure was selected due to its excellent psychometric properties, including validation on adult populations, and identification of motives relevant to our research questions [25, 29, 30]. Due to financial constraints, single items were chosen to represent the drinking motives. Single items have been used elsewhere to capture alcohol drinking motives, including motives captured in the DMQ‐R [41, 42]. Due to evidence that coping‐anxiety and coping‐depression motives are differentially associated with drinking patterns and socioeconomic status [20, 37] we selected two items from the coping subscale which represent these different aspects of coping. While the modified DMQ‐R already distinguishes between these two motives, its authors note it has some unsatisfactory psychometric properties and has not yet been validated on adults [20], therefore, we chose to use items from the DMQ‐R [19].

Item selection was informed by each item's psychometric properties and personal and public involvement and engagement (PPIE). The selected items were:

- Because it gives you a pleasant feeling (Enhancement).
- Because it makes social gatherings more fun (Social).
- To fit in with a group that you like (Conformity).
- Because you feel more self‐confident and sure of yourself (Coping–anxiety).
- To forget about your problems (Coping–depression).

Responses were recorded on a 5‐point scale (1 = Never/Almost Never, 2 = Some of the time, 3 = Half of the time, 4 = Most of the time, 5 = Almost Always/Always) and treated as continuous variables.

#### 2.3.3 Harmful Alcohol Consumption

The Alcohol Use Disorders Identification Test‐C (AUDIT‐C) measured hazardous alcohol consumption [40], discriminating between those at higher or lower risk of alcohol‐related harm. A three‐item scale captures frequency of alcohol consumption, typical numbers of units consumed during a drinking occasion, and frequency of heavy episodic drinking (6 or more units of alcohol in a single drinking occasion). Responses were recoded to correspond with validated AUDIT‐C scoring to produce a total score between 0 and 12, treated as a continuous variable. Non‐drinkers were excluded; therefore, scores in the study sample ranged from 1 to 12.

#### 2.3.4 Sociodemographic Variables

The routinely collected variables in the ATS that were used in the analysis included:

– Age (16–24, 25–34, 35–44, 45–54, 55–64, 65+ years);
– Gender (male, female);
– Highest level of education attained, categorised as: secondary education (or equivalent); pre‐university qualification (e.g., A‐levels, international baccalaureate diploma, or equivalent); bachelor's degree (or equivalent undergraduate degree); and postgraduate degree (e.g., Master's, PhD, or equivalent);
– Social grade based on profession (AB = higher/intermediate managerial, administrative or professional, C1 = supervisory, clerical and junior managerial, administrative or professional, C2 = skilled manual workers, DE = semi‐skilled and unskilled manual workers, pensioners, casual and lowest grade workers, unemployed and in receipt of state benefits only [43]).

Age, social grade, and education were treated as ordinal variables. Social grade was coded so that a higher value denoted a higher social grade. Ethnicity is reported descriptively (White, Black, Asian, Mixed heritage, other, Table 1), but not included in the path analysis.

**TABLE 1:** Sample characteristics (weighted, *n* = 2591).

Characteristic	Statistic
NoLo consumption	*n* (%)
At least monthly	547 (21.1)
Less than once a month	2044 (78.9)
Alcohol drinking motives^a^	Mean (95% CI)
Enhancement	2.71 (2.60, 2.82)
Social	2.64 (2.53, 2.75)
Conformity	1.60 (1.49, 1.71)
Coping‐anxiety	1.60 (1.49, 1.71)
Coping‐depression	1.30 (1.19, 1.41)
Hazardous alcohol consumption	Mean (95% CI)
AUDIT‐C score	4.36 (4.25, 4.67)
AUDIT‐C score risk classifications	*n* (%)
Low risk (score 0–4)	1548 (59.7%)
Increasing risk (score 5–7)	685 (26.4%)
Higher risk (score 8–10)	320 (12.4%)
Possible dependence (score 11–12)	38 (1.5%)
Age, years	*n* (%)
16–24	306 (11.8%)
25–34	414 (16.0%)
35–44	439 (17.0%)
45–54	453 (17.5%)
55–64	435 (16.8%)
65+	545 (21.0%)
Gender	*n* (%)
Male	1321 (51.0%)
Female	1270 (49.0%)
Social grade	*n* (%)
AB (higher or intermediate managerial)	758 (29.3%)
C1 (supervisory/clerical, junior managerial administrative/professional)	808 (31.2%)
C2 (skilled manual)	542 (20.9%)
DE (semi−/un‐skilled manual, casual or lowest grade, pensioners, others who depend on the welfare state for their income).	483 (18.6%)
Education	*n* (%)
Secondary school/equivalent	668 (25.8%)
College (A Levels)/equivalent	668 (25.8%)
Undergraduate degree/equivalent	821 (31.7%)
Postgraduate degree/equivalent	434 (16.7%)
Ethnicity	*n* (%)
White British/other	2322 (89.6%)
Black British/other	96 (3.7%)
Asian British/other	65 (2.5%)
Mixed heritage	62 (2.4%)
Other ethnicities including not specified	46 (1.8%)

Abbreviations: AUDIT, Alcohol Use Disorder Identification Test; CI, confidence interval; NoLo, alcohol‐free and low‐alcohol.

^*a*^ Alcohol drinking motives are scored on a 5‐point scale 1 = Never/Almost never, 5 = Always/Almost always.

### 2.4 Personal and Public Involvement and Engagement

The Alcohol Toolkit Study is a well‐established survey, therefore the PPIE focused on the selection of questions to capture alcohol drinking motives. Shortlisted items from the DMQ‐R were presented to seven members of the Stirling Food and Alcohol Discussion Group (https://spectrum.ed.ac.uk/about/public‐involvement). The group discussed these items, expressing their level of agreement with the selected items. An alternative to the shortlisted item for conformity was suggested. This item had good factor loadings and face‐validity; therefore, the shortlisted item was updated to reflect the views of the PPIE group. Further details regarding our PPIE work is described elsewhere [34].

### 2.5 Ethics

Ethics approval for data collection was obtained by The University College London, who has overall ownership of the Alcohol Toolkit Study (ID 0498/001). Trained interviewers obtain verbal informed consent from participants prior to undertaking the survey.

### 2.6 Pre‐Registration

The study's analytical plan was pre‐registered on the Open Science Framework (osf.io/6rn3w). This outlines the path analysis reported in this paper and the cross‐sectional study exploring direct associations which has been published separately [34]. Results from other published work, including our earlier work investigating direct pathways [9, 34] led to the following changes:

- Social grade and education were included as observed variables, rather than producing a latent variable for socioeconomic status. During preparatory work to construct the latent variable, inconsistent relationships between social grade, education and index of multiple deprivation [44] with NoLo consumption were observed. Therefore, it did not make conceptual sense to construct a latent variable for socioeconomic status in this analysis.
- A total score was used for AUDIT‐C instead of a latent variable, to align with common practice [40, 45].
- Analyses were population weighted.
- Pathways between alcohol drinking motives and NoLo were explored via hazardous drinking. This was in acknowledgement of the findings from the earlier analyses [34] and further development of the conceptual model.
- The statistical significance of pathways is inferred through bootstrapped confidence intervals rather than *p* values [36].

### 2.7 Analysis

Data preparation and analyses were undertaken in R 4.3.1, an open access software package [46]. The following variables had missing data: gender, *n* = 6; social grade, *n* = 106. Missing data from the social grade variable was replaced with C1, as is common practice with this data and produces similar estimates [47, 48]. This created a complete dataset for 2549 out of a possible 2555 (99.8%). We proceeded using complete cases, which were population weighted.

Path analysis was undertaken using ‘lavaan’ [49]. Path analysis is a type of structural equation modelling that does not use latent variables [36]. The method incorporates mediating and moderating variables that capture indirect pathways between observed factors. The robust version of weighted least squares was used as the model estimator, with scaled fit statistics and both standardised and unstandardised coefficients reported for the selected model. This is appropriate when there are ordinal and categorical variables [36].

Multivariate normality was assessed using Mahalanobis distance [50]. Fifty‐four outliers were identified. Their exclusion did not influence the model results (see Table 2 and Tables S1–S5); therefore, the original sample was retained.

**TABLE 2:** A comparison of global fit statistics.

Model	*X*2	df	*p*	CFI	TLI	RMSEA (90% CI)	SRMR
Recommended fit thresholds [43]			n.s.	> 0.95	> 0.95	< 0.06	< 0.05
1. Direct pathways	34.668	1	< 0.001	0.968	0.334	0.115 (0.084, 0.149)	0.000
2. Direct pathways—outliers removed	62.192	1	< 0.001	0.938	−0.310	0.157 (0.125, 0.191)	0.000
3. Fully mediated	16.871	20	0.661	1.000	1.003	0.000 (0.000, 0.014)	0.007
**4. Selected mediated model**	**18.582**	**18**	**0.418**	**0.999**	**0.999**	**0.004 (0.000, 0.018)**	**0.008**
5. Mediated—outliers removed	17.745	18	0.473	1.000	1.000	0.000 (0.000, 0.018)	0.007

*Note:* The selected model is in bold.

Abbreviations: CFI, comparative fit index; CI, confidence interval; RMSEA, root mean square error of approximation; SRMR, standardised root mean residual; TLI, Tucker‐Lewis Index.

During the first phase of model building, direct pathways between age, gender, social grade, education, alcohol drinking motives, hazardous drinking and NoLo consumption were explored. Age, gender, social grade and education were exogenous variables in the model. Global model fit was poor (Table 2). Direct pathways *a* < 0.1 were retained in the model (Table S1).

**FIGURE 2:**
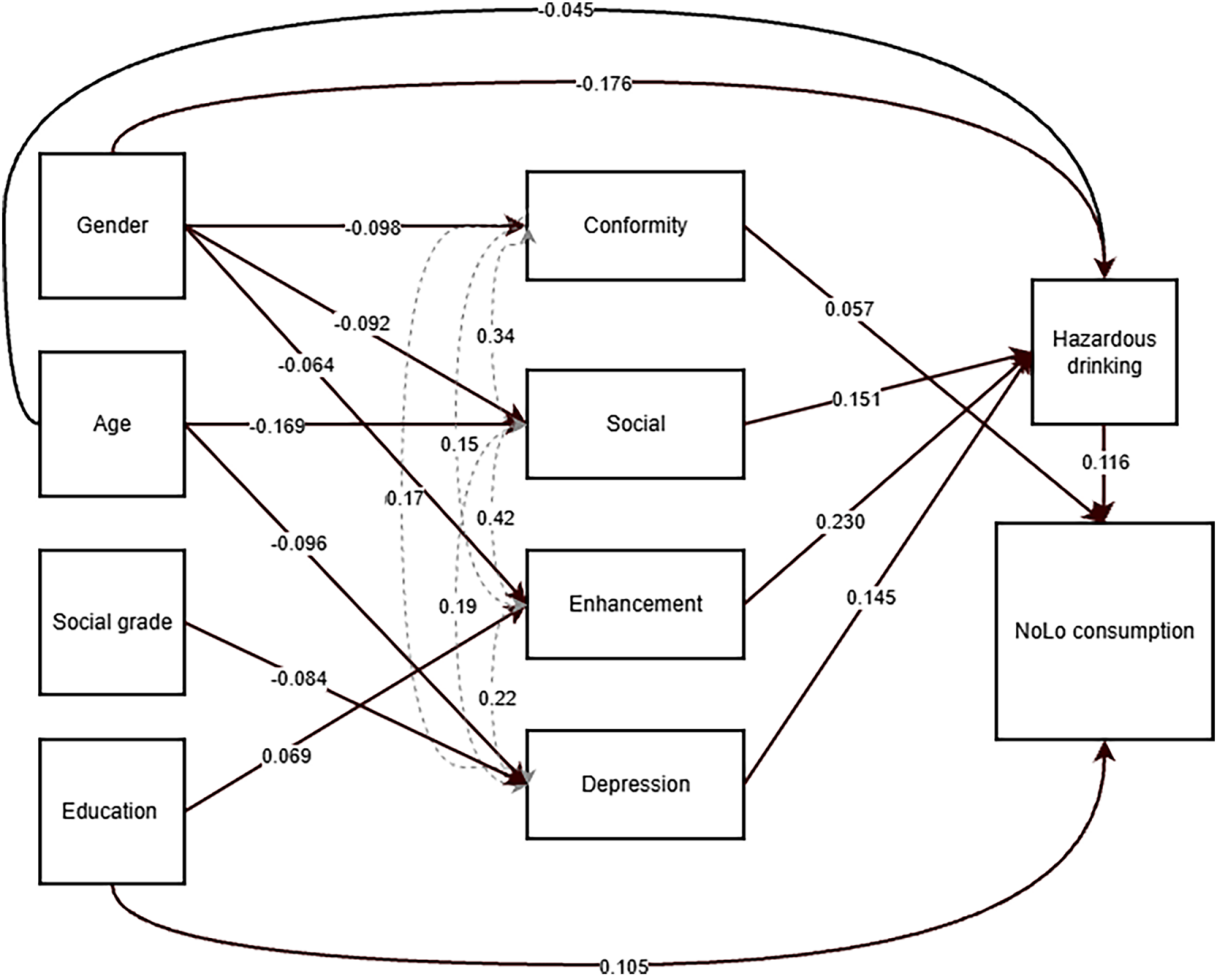
The relationship between sociodemographic characteristics and NoLo consumption via drinking motives and hazardous drinking. 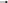 : Pathways reporting standardised coefficients. 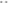 : Covariance. *Note:* The reference category for gender is male.

In the second phase, mediated models were built. In the first mediated model, hazardous drinking and the alcohol drinking motives that had significant pathways ‘in’ and ‘out’ identified from the first model were assessed for mediation effects considering our directional and non‐directional hypotheses (Table 2). Pathways with bootstrapped 95% CI that did not cross zero were retained, and a more parsimonious model was evaluated (Table 3, Figure 2), which had excellent model fit. Global fit statistics are presented for all models (Table 2). Local fit statistics for the selected model are presented in the main paper (Table 3), while local fit statistics for the preceding models are available in the Supporting Information (S1–S5) [36].

**TABLE 3:** Direct and indirect pathways to NoLo consumption identified in the selected model.

	Standardised estimate *β*	Unstandardised estimate *b*	Bootstrapped results	
			SE	95% CI
Direct pathways to NoLo				
Education ➔ NoLo	0.105	0.102	0.033	0.047, 0.174
Hazardous drinking ➔ NoLo	0.116	0.046	0.013	0.026, 0.076
Conformity ➔ NoLo	0.057	0.053	0.028	0.010, 0.119
Indirect pathways to NoLo				
1. Social grade ➔ Depression ➔ Hazardous drinking ➔ NoLo	−0.001	−0.001	0.000	−0.002, −0.000
2. Gender^a^ ➔ Conformity ➔ NoLo	−0.006	−0.011	0.006	−0.026, −0.002
3. Gender^a^ ➔ Hazardous drinking ➔ NoLo	−0.021	−0.041	0.012	−0.068, −0.021
4. Gender^a^ ➔ Social ➔ Hazardous drinking ➔ NoLo	−0.002	−0.003	0.001	−0.006, −0.001
5. Gender^a^ ➔ Enhancement ➔ Hazardous drinking ➔ NoLo	−0.002	−0.003	0.001	−0.006, −0.000
6. Age ➔ Hazardous drinking ➔ NoLo	−0.039	−0.003	0.002	−0.009, −0.001
7. Age ➔ Social ➔ Hazardous drinking ➔ NoLo	−0.003	−0.002	0.001	−0.003, −0.001
8. Age ➔ Depression ➔ Hazardous drinking ➔ NoLo	−0.002	−0.001	0.000	−0.002, −0.000
9. Education ➔ Enhancement ➔ Hazardous drinking ➔ NoLo	0.002	0.002	0.001	0.001, 0.004
Total indirect pathways	−0.028	−0.050	0.014	−0.079, −0.024
Total direct and indirect pathways	0.250	0.151	0.042	0.084, 0.251

Abbreviations: CI, confidence interval; NoLo, alcohol‐free and low‐alcohol.

^a^ Male is the reference category.

## 3 Results

### 3.1 Demographic Characteristics

The study sample is summarised in Table 1. Just over one‐fifth of respondents reported drinking alcohol‐free (< 0.05) or low‐alcohol (≤ 1.2% ABV, NoLo) drinks at least monthly. Drinking for enhancement (Mean = 2.71, SD = 1.44, 95% CI 2.60, 2.82) and for social reasons (Mean = 2.64, SD = 1.38, 95% CI 2.53, 2.75) were the most strongly endorsed reasons for drinking alcohol. Drinking to cope with depression was the least endorsed (Mean = 1.30, SD = 0.81, 95% CI 1.19, 1.41).

The scaled fit indices for the selected model indicated excellent model fit (Table 2) [51]. In line with our pre‐registered analysis plan, the coping‐anxiety motive was removed as it did not explain NoLo consumption in the model (Tables S1–S3). The model explained 2.96% of the variance in monthly NoLo consumption (*R* ^2^ = 0.03). The standardised total effect (direct plus indirect pathways) on NoLo consumption was moderate and positive (*β* = 0.255, Table 3). This was slightly attenuated by a small negative standardised total indirect effect (*β* = −0.028). This attenuation resulted from the serial mediating variables (drinking motives) reducing the strength of the positive relationship between hazardous drinking and NoLo consumption. A full table of results including model covariances, thresholds and intermediate pathways for the selected model is also presented in the Supporting Information (Table S1).

### 3.2 Model Overview

Local model statistics for the selected model are presented in Figure 2 and Table 3. Figure 2 illustrates the model pathways, presenting standardised path coefficients between each direct relationship captured in the model. The overall path coefficients for all direct and indirect pathways are presented in Table 3.

One direct pathway between sociodemographic characteristics and NoLo consumption was identified. Higher levels of education were positively associated with regular NoLo consumption (*β* = 0.105, 95% CI [0.047, 0.174], Table 3).

#### 3.2.1 Pathways Using Drinking Motives to Explain Sociodemographic Differences in NoLo Consumption

Nine indirect pathways were found between the sociodemographic characteristics and NoLo consumption, seven of which included alcohol drinking motives.

#### 3.2.2 Primary Hypothesis: Drinking to Cope With Depression Mediates the Relationship Between Social Grade and NoLo Consumption

Drinking to cope with depression acted as a serial mediator between social grade and NoLo consumption through hazardous drinking. While those who reported higher AUDIT‐C scores were *overall* more likely to report drinking no/lo at least monthly, this association diminished for respondents who regularly drank alcohol to cope with depression, who were more often from lower social grades. These associations counteracted one another, producing a negligible negative path coefficient (Indirect pathway 1: *b* = −0.001, 95% CI [−0.002, −0.000], Table 3). This finding supports the hypothesis that drinking alcohol to cope with depression acts as a barrier preventing hazardous drinkers from lower social grades transitioning to NoLo consumption.

#### 3.2.3 The Mediating Role of Alcohol Drinking Motives and Hazardous Drinking in Explaining Relationships Between Gender, Age, Education and NoLo Consumption

##### 3.2.3.1 Gender and NoLo Consumption

While no overall association between gender and NoLo was observed (Table S1), this relationship was mediated by conformity, enhancement and social drinking motives, as well as hazardous drinking. Women were less likely to report hazardous drinking, and this decreased likelihood indirectly contributed to their being less likely to drink NoLo products (Indirect pathway 3: *b* = −0.041, 95% CI [−0.068, −0.021]).

Women were less likely to drink alcohol to conform, a motive positively directly associated with NoLo consumption. This partially mediated pathway found being a woman (relative to a man), through the mechanism of drinking alcohol to conform, was associated with a negative indirect effect (Indirect pathway 2: *b* = −0.011, 95% CI [−0.026, −0.002]), this finding indicating that men who drank to conform were more likely to drink NoLo products. These effects were counteracted by enhancement and social drinking motives. Men were more likely to endorse these motives, which were positively associated with hazardous drinking (Figure 2, note: men are the reference therefore the coefficient is negative). These motives dampened the strong relationship between hazardous drinking and NoLo consumption, indicating serially partially mediated pathways where men whose alcohol consumption was driven by enhancement and social motives were less likely to consume NoLo drinks than other heavy drinkers (Indirect pathway 4: *b* = −0.003, 95% CI [−0.006, −0.001], Indirect pathway 5: *b* = −0.003, 95% CI [−0.009, −0.001]). These complex relationships help explain why the total effect of gender on NoLo consumption was negligible.

##### 3.2.3.2 Age and NoLo Consumption

No overall association between age and NoLo was observed. This relationship was, however, mediated by coping‐depression and social drinking motives and hazardous drinking. Hazardous drinking partially mediated the relationship between age and NoLo consumption. Younger respondents who drank at more hazardous levels were more likely to report NoLo products (Indirect pathway 6: *b* = −0.003, 95% CI [−0.009, −0.001], Table 3).

Younger respondents were more likely to report drinking alcohol to cope with depression and for social reasons, evidenced by their inverse relationships (Figure 2). These motives partially serially mediated the age ➔ NoLo relationship via hazardous drinking. The positive association between hazardous drinking and NoLo consumption was again diminished for participants reporting coping‐depression or enhancement motives, who were typically younger, resulting in negligible coefficients for these pathways (Indirect pathway 7: *b =* −0.002, 95% CI [−0.003, −0.001], Indirect pathway 8: *b* = −0.001, 95% CI [−0.002, −0.000]). The overall lack of a direct age effect appears to be a result of competing indirect pathways: the increased adoption of NoLo consumption among younger hazardous drinkers is offset by the dampening effect of the coping and enhancement motives that are also prevalent in this younger cohort.

##### 3.2.3.3 Education and NoLo Consumption

In addition to a direct positive association between education and NoLo consumption, a partially mediated pathway was identified. More educated respondents were more likely to report drinking alcohol for enhancement, which was associated with hazardous drinking but weakened the relationship between hazardous drinking and NoLo consumption for this group (Indirect pathway 9: *b* = 0.002, 95% CI [0.001, 0.004], Table 3).

## 4 Discussion

Our study provides insight into the relationships between sociodemographic factors, alcohol drinking motives, hazardous drinking and NoLo consumption, explaining 3% of the variance in at least monthly NoLo consumption. The results highlight the importance of understanding sociodemographic patterning of behaviour, even when there appears to be no overall relationship between a characteristic and an outcome. We identified seven pathways where alcohol drinking motives mediated relationships between sociodemographic variables and NoLo consumption, six of which involved serial mediation via hazardous drinking (Figure 2). The path coefficients indicated significant yet complex relationships.

With regards to our primary hypothesis that drinking to cope with depression would mediate the relationship between social grade and NoLo consumption, we found evidence of a small serial mediation effect. In line with previous research [16, 17], respondents from lower social grades were more likely to drink alcohol to cope, which was positively associated with hazardous drinking. Notably, these hazardous drinkers, unlike hazardous drinkers overall, were *not* more likely to consume NoLo drinks. While no other relationships between social grade and NoLo consumption were observed, the negligible pathway coefficient for social grade ➔ coping ➔ hazardous drinking ➔ NoLo consumption suggests that other unobserved factors associated with social grade may be important in explaining the positive relationship between social advantage and NoLo consumption. These are discussed further below.

Our non‐directional hypotheses exploring the potential mediating effects of alcohol drinking motives on relationships between sociodemographic characteristics and NoLo consumption demonstrated that the absence of direct relationships between age, gender and education could be partly understood through several mediating pathways working in opposing directions, masking any total effect.

Specifically, our findings suggest that for hazardous drinkers, the relationship between hazardous drinking and NoLo consumption is weakened when alcohol consumption is driven by enhancement, social reasons, or coping with depression motives. NoLo drinks are poor substitutes for alcohol for those whose behaviour is driven by the psychoactive elements of alcohol [29, 32]. This suggests individuals drinking for these reasons should be less likely to perceive NoLo drinks as a satisfactory alternative. Conversely, the conformity motive increased the likelihood of consumption. This dichotomy aligns with the qualitative findings that NoLo products are appreciated for their ability to enable consumers to conform to our social rituals, yet rejected by those who drink alcohol for its inebriating effect [10, 27, 28, 29, 30, 31, 32].

These complex relationships present differently across the sociodemographic characteristics explored in this model. The relationship between gender and NoLo consumption was mediated by drinking motives acting in opposing directions. Males who drank to conform were associated with an increased likelihood of regular NoLo consumption, consistent with existing evidence that NoLo drinks facilitate this strategy [27, 29, 30, 31, 52, 53]. However, the positive associations between men ➔ hazardous drinking ➔ NoLo consumption were counteracted by those men who drank heavily for enhancement or social motives. These groups of men were *less likely* to consume NoLo drinks compared to other men drinking at similar hazardous levels.

Younger respondents were more likely to drink alcohol for social reasons and to cope with depression, aligning with findings from a recent cross‐cultural study [54]. Consequently, while younger individuals engaging in hazardous drinking were overall more likely to consume NoLo, this tendency appeared reduced among those whose hazardous drinking was driven by social or coping motives.

Finally, while the level of education was directly positively associated with NoLo consumption, individuals with higher level qualifications were more likely to drink for enhancement, which was correlated with hazardous drinking. As previously described, heavier drinkers who drank for enhancement exerted a negative influence on the likelihood of NoLo consumption.

In summary, our findings illustrate the complex role of alcohol drinking motives in explaining NoLo consumption, particularly regarding its relationship with hazardous drinking. The results are conceptually appealing: there is a growing qualitative literature in the UK which describes the advantages of NoLo products being in their ability to replicate the sensory experience of drinking alcohol, i.e., the look, taste and even process of pouring a drink into a glass specifically for that purpose [27, 31, 32, 53], whereas for heavier drinkers whose drinking is driven by a desire for inebriation, or as a coping strategy, NoLo drinks appear a less acceptable alternative [32, 35].

We should note however that the lower uptake of NoLo in less socially advantaged groups appears only partly explained by drinking motives. Further work should explore structural and contextual barriers to uptake. The wider literature indicates that the price of these products and perceived value for money may be important [32, 35]. However, it is important to be mindful of what NoLo drinks are being compared to, with consumers often perceiving NoLos as poor value compared to *soft drinks* rather than standard alcoholic beverages [35, 55]. The taste of NoLo products has been identified as a factor that may encourage or discourage consumption [12, 56], with those who have less disposable income potentially being less willing to pay for a novel product to discover they don't like the taste.

Alcohol and NoLo consumption must be understood within the broader contexts of individuals' lives and the weight they place on their short and long‐term health and well‐being. For some groups, particularly those who are younger and less affluent, the opportunity cost of experiencing the immediate negative aspects of alcohol consumption may be lower due to fewer competing engagements and responsibilities that require an unimpaired state [57, 58, 59]. Socially advantaged groups typically have higher levels of health literacy and engagement in their own health and well‐being, which may lead to these groups proactively substituting alcohol for NoLo alternatives as part of a healthy lifestyle which embraces moderation, rather than in response to harmful drinking [58, 59].

Whether the overall positive association between hazardous drinking and NoLo consumption will improve public health requires further scrutiny using studies that explicitly explore substitution effects. While modest in size, our findings that coping, social and enhancement motives weaken this relationship is concerning, given that these effects are likely to be diluted in this study where not all NoLo consumption will have been a substitution for standard alcoholic drinks. Drinking for these reasons is associated with heavier drinking and alcohol harm [60], therefore, it is necessary to prioritise strategies which reduce harm amongst these groups.

Where NoLo drinks appear to be more successful is in providing a lower‐risk alternative for those drinking to conform to social pressures prevalent in UK society [61]. However, this may be a short‐term benefit if it is perpetuating the normalisation of alcohol in our culture.

This study was conducted when NoLo products comprise a small but expanding proportion of the UK market. It is not unfeasible that sociodemographic trends shift as awareness of these products increases. We may see a decrease in socioeconomic variability through social diffusion, where NoLo drinks become normalised amongst a wider range of settings [62], in the same way we have seen the trend filter through from younger to older age groups [14].

### 4.1 Strengths and Limitations

This is the first study to explore whether alcohol drinking motives help explain sociodemographic patterns of NoLo consumption. This is important when understanding the potential benefit of policies which promote these products to identify where additional support will be needed, particularly regarding reducing health inequalities. The study was well‐powered and capable of detecting small effect sizes, meaningful at a population level. The data is taken from a representative sample and population weighted, using items from a validated drinking motives questionnaire that correspond with the motives that appear pertinent for these products. The current study explored consumption of all NoLo products, and therefore should be more applicable to a wider population than studies which have explored single NoLo beverages, e.g., non‐alcoholic beers [10, 11, 12, 13].

The data is taken from a cross‐sectional sample, so we cannot infer causation. This model presents at least monthly NoLo consumption as the dependent variable. As discussed previously, this will include not only those who are drinking alcohol as a substitute but those who are drinking NoLo products as an alternative to other non‐alcoholic drinks, for example when pregnant or driving. This may have weakened the observed effects for alcohol drinking motives. We were unable to create a latent variable for socioeconomic status; therefore, we use education and social grade as proxies to capture social advantage. These measures are widely used and useful for enabling comparative work; however, there are limitations regarding social grade's external validity, as it classifies students and those in receipt of pensions in the lowest category.

Due to resource constraints, we were unable to use the full DMQ‐R or the DMQ‐R SF [19, 22]. While standard practice for large surveys, where the constructs of interest comprise a small aspect of the total survey [63], it may nonetheless impede the validity and reliability of the study findings, by not wholly capturing the corresponding dimension. We aimed to mitigate this limitation through our procedure for selecting representative items, as described in the methods. We were reassured that the patterns of endorsement for our selected items are consistent with a recently conducted, cross‐national study of drinking motives (including Great Britain), supporting the reliability of our estimates [54], and many of the relationships between sociodemographic characteristics, drinking motives and alcohol consumption replicated previously established patterns [64]. However, we recommend further work is conducted using the full DMQ‐R measure [19], or other validated measure of adult drinking motives (e.g., [21]).

## 5 Conclusions

This work has provided meaningful insights into the relationships between measures of socioeconomic status, age, gender and NoLo consumption, which is particularly relevant in the context of the UK's public health interest in promoting NoLo drinks. Drinking to cope with depression, more common amongst lower social grades, was found to weaken the relationship between hazardous drinking and NoLo consumption. Drinking for enhancement and social reasons, more common amongst men, younger groups and the more educated, also diminished this relationship. While there is potential for benefit from promoting NoLo drinks to people who are drinking to conform, those drinking alcohol for enhancement, for social reasons, or to cope with depression may be less likely to substitute alcohol with NoLo drinks. The amount of variance unexplained in the model indicates that other unmeasured factors contribute to NoLo consumption behaviour. Factors worthy of future consideration include: additional motives not captured in the DMQ‐R such as taste or ritual [21, 42], the reasons why people are choosing *not* to drink alcohol, and motives *for* choosing NoLo products opposed to other non‐alcoholic alternatives [27, 32]. Future research will benefit from explicitly capturing substitution effects.

Further work should aim to validate these insights by replicating these findings in different contexts and amongst different populations, particularly those at increased risk of alcohol‐related harm, such as LGBTQ+ groups, racial/ethnic minority groups, or those with co‐occurring mental health conditions [36], particularly if this market continues to grow. It will be worth repeating this study with a more sensitive measure of consumption than a binary variable capturing less than/at least monthly consumption. Furthermore, as jurisdictions vary in their definitions of alcohol‐free and particularly low‐alcohol, separating these two drink types may add value. Finally, a more systemic model capturing the influence of price and availability will be of particular use for those working in public health wishing to maximise the effectiveness of alcohol policies amongst those most at risk of harm.

## Author Contributions

**Lucy Burke:** conceptualisation (lead), data curation (lead), methodology (lead), project administration (lead), formal analysis (lead), writing – original draft (lead), writing – review and editing (lead). **Colin Angus:** conceptualisation (supporting), methodology (supporting), formal analysis (supporting), writing – review and editing (supporting). **Jamie Brown:** data curation (supporting), methodology (supporting), formal analysis (supporting), writing – review and editing (supporting). **Inge Kersbergen:** conceptualisation (supporting), methodology (supporting), project administration (supporting), formal analysis (supporting), writing – review and editing (supporting). Each author certifies that their contribution to this work meets the standards of the International Committee of Medical Journal Editors.

## Funding

This study was funded by a Wellcome Trust PhD grant (218462/Z/19/Z, grant recipient LB). Overall Alcohol Toolkit data collection is funded by Cancer Research UK (grant ref.: PRCRPG‐Nov21\100002) in England and the UK Prevention Research Partnership (via SPECTRUM, grant ref.: MR/S037519/1) in Scotland and Wales. The collection of NoLo consumption frequency data was funded by the NIHR Public Health Research programme (NIHR135310). The collection of alcohol drinking motives data was funded by Wellcome (218462/Z/19/Z). The funders were not involved in the study design, data collection and analysis, decision to publish, or preparation of the manuscript. The views expressed are those of the author(s) and not necessarily those of Wellcome, Cancer Research UK, NIHR or the Department of Health and Social Care.

## Consent

The authors have nothing to report.

## Conflicts of Interest

L.B., I.K. and J.B. have received funding for ongoing, unrelated research on alcohol‐free and low‐alcohol drinks from Alcohol Change UK (ACUK), which received < 0.6% of its funds in 2024–2025 from Lucky Saint, an organisation that produces and sells non‐alcoholic drinks, and owns a pub that sells standard alcoholic drinks. In March 2025, Lucky Saint became an associate member of The Portman Group, a self‐regulatory organisation that is fully funded and controlled by the alcohol industry. ACUK has a strict policy of not accepting any funds from, nor being subject to any influence whatsoever from, the alcohol industry, including through its investment portfolio. ACUK has stated that it is in full compliance with its policy. ACUK did not fund the present research and had no role in any stage of this work. The other author declares no conflicts of interest.

## Supporting information


**Figure S1:** Participant recruitment and inclusion in the study.
**Table S1:** Parameter Estimates for model 1 including direct effects only.
**Table S2:** Model parameter estimates for direct model with outliers removed.
**Table S3:** Fully mediated model (bootstrapped).
**Table S4:** Selected Mediated Model.
**Table S5:** Mediated model with outliers removed.
**Table S6:** The relationship between hazardous drinking and NoLo consumption without drinking motives included in the model.

## Data Availability

Study syntax are openly available at: https://github.com/LucyCBurke/nolo_dm_path. Non‐identifiable anonymised data used in the analysis may be available on request. Please direct any enquiries to the study authors.
