# Supplementary material for: Do the Reasons People Drink Alcohol Aid Our Understanding of Sociodemographic Differences in Alcohol‐Free and Low‐Alcohol Consumption? A Path Analysis on a Cross‐Sectional Study of Adult Alcohol Drinkers in Great Britain
Source: Drug Alcohol Rev. 2026 May 5;45:e70159. doi: 10.1111/dar.70159 (PMC13139994; doi:10.1111/dar.70159)
Supplement: Supplementary file 1 — Figure S1: Participant recruitment and inclusion in the study. Table S1: Parameter Estimates for model 1 including direct effects only. Table S2: Model parameter estimates for direct model with outliers removed. Table S3: Fully mediated model (bootstrapped). Table S4: Selected Mediated Model. Table S5: Mediated model with outliers removed. Table S6: The relationship between hazardous drinking and NoLo consumption without drinking motives included in the model. [file DAR-45-0-s001.docx]

**SUPPORTING INFORMATION**

**FIGURE S1. Participant recruitment and inclusion in the study**

Eligible for analysis (*n*=2555)

Sample using complete cases (*n*=2549)

Excluded due to ineligibility

- Did not drink alcohol in previous 12 months (*n*=1169)

Missing data

- Gender (*n*=6)
- Social grade (n=106), replaced with C1.

Excluded due to data reporting

- Inconsistent reporting of NoLo (*n*=163)
- Did not know whether they drank for a particular motive:

Enhancement (*n*=64)

Social (*n*=79)

Conformity (*n*=66)

Anxiety (*n*=77)

Depression (*n*=41)

- Described gender in another way (*n*=13)

Assessed for eligibility (*n*=4089)

Assessed for data quality/ sufficient response rates within category (*n*=2920)

**Table S1. Parameter Estimates for model 1 including direct effects only**

|  |  |  |  |  |  | **95% CI** | |  |  |
| --- | --- | --- | --- | --- | --- | --- | --- | --- | --- |
| **Dependent variable** | **Predictor variable** | **Unstandardised coefficient** | **Standard error** | **z** | **P value** | **lower** | **upper** | **Standardised coefficient** | **Partially standardised coefficient** |
| **Regressions** |  |  |  |  |  |  |  |  |  |
| At least monthly NoLo consumption | Level of education | 0.105 | 0.032 | 3.271 | 0.001 | 0.042 | 0.168 | 0.108 | 0.104 |
| At least monthly NoLo consumption | AUDIT C | 0.042 | 0.014 | 2.991 | 0.003 | 0.014 | 0.069 | 0.105 | 0.105 |
| At least monthly NoLo consumption | Gender | -0.075 | 0.063 | -1.188 | 0.235 | -0.200 | 0.049 | -0.037 | -0.075 |
| At least monthly NoLo consumption | Social | -0.036 | 0.026 | -1.401 | 0.161 | -0.087 | 0.014 | -0.050 | -0.050 |
| At least monthly NoLo consumption | Enhancement | 0.022 | 0.024 | 0.898 | 0.369 | -0.025 | 0.069 | 0.031 | 0.031 |
| At least monthly NoLo consumption | Depression | -0.007 | 0.044 | -0.170 | 0.865 | -0.093 | 0.078 | -0.006 | -0.006 |
| At least monthly NoLo consumption | Conformity | 0.055 | 0.030 | 1.849 | 0.064 | -0.003 | 0.114 | 0.058 | 0.058 |
| At least monthly NoLo consumption | Anxiety | 0.049 | 0.034 | 1.416 | 0.157 | -0.019 | 0.116 | 0.050 | 0.050 |
| At least monthly NoLo consumption | Social grade | 0.044 | 0.035 | 1.271 | 0.204 | -0.024 | 0.111 | 0.047 | 0.043 |
| At least monthly NoLo consumption | Age | -0.004 | 0.019 | -0.186 | 0.852 | -0.042 | 0.035 | -0.006 | -0.004 |
| AUDIT C | Gender | -0.909 | 0.105 | -8.688 | 0.000 | -1.114 | -0.704 | -0.179 | -0.357 |
| AUDIT C | Social grade | 0.090 | 0.056 | 1.613 | 0.107 | -0.019 | 0.198 | 0.038 | 0.035 |
| AUDIT C | Level of education | -0.097 | 0.053 | -1.809 | 0.070 | -0.201 | 0.008 | -0.040 | -0.038 |
| AUDIT C | Conformity | -0.077 | 0.051 | -1.512 | 0.131 | -0.177 | 0.023 | -0.032 | -0.032 |
| AUDIT C | Enhancement | 0.402 | 0.036 | 11.173 | 0.000 | 0.331 | 0.472 | 0.227 | 0.227 |
| AUDIT C | Depression | 0.476 | 0.064 | 7.467 | 0.000 | 0.351 | 0.601 | 0.152 | 0.152 |
| AUDIT C | Social | 0.318 | 0.040 | 7.989 | 0.000 | 0.240 | 0.396 | 0.172 | 0.172 |
| AUDIT C | Age | -0.054 | 0.032 | -1.683 | 0.092 | -0.116 | 0.009 | -0.035 | -0.021 |
| AUDIT C | Anxiety | 0.011 | 0.049 | 0.233 | 0.816 | -0.085 | 0.108 | 0.005 | 0.005 |
| Conformity | Gender | -0.208 | 0.048 | -4.300 | 0.000 | -0.302 | -0.113 | -0.097 | -0.194 |
| Conformity | Level of education | -0.011 | 0.024 | -0.458 | 0.647 | -0.058 | 0.036 | -0.011 | -0.010 |
| Conformity | Social grade | -0.027 | 0.025 | -1.078 | 0.281 | -0.077 | 0.022 | -0.027 | -0.025 |
| Conformity | Age | -0.001 | 0.014 | -0.060 | 0.952 | -0.028 | 0.027 | -0.001 | -0.001 |
| Enhancement | Gender | -0.184 | 0.064 | -2.901 | 0.004 | -0.309 | -0.060 | -0.064 | -0.128 |
| Enhancement | Level of education | 0.107 | 0.033 | 3.258 | 0.001 | 0.043 | 0.171 | 0.078 | 0.074 |
| Enhancement | Social grade | 0.027 | 0.034 | 0.786 | 0.432 | -0.040 | 0.093 | 0.020 | 0.019 |
| Enhancement | Age | 0.025 | 0.020 | 1.275 | 0.202 | -0.013 | 0.063 | 0.029 | 0.017 |
| Depression | Social grade | -0.068 | 0.021 | -3.260 | 0.001 | -0.109 | -0.027 | -0.090 | -0.084 |
| Depression | Gender | 0.030 | 0.039 | 0.764 | 0.445 | -0.046 | 0.106 | 0.018 | 0.036 |
| Depression | Level of education | -0.020 | 0.021 | -0.967 | 0.333 | -0.060 | 0.020 | -0.026 | -0.024 |
| Depression | Age | -0.083 | 0.010 | -7.985 | 0.000 | -0.104 | -0.063 | -0.171 | -0.102 |
| Anxiety | Social grade | -0.065 | 0.025 | -2.564 | 0.010 | -0.115 | -0.015 | -0.067 | -0.062 |
| Anxiety | Gender | 0.024 | 0.049 | 0.480 | 0.631 | -0.073 | 0.120 | 0.011 | 0.023 |
| Anxiety | Level of education | 0.005 | 0.026 | 0.193 | 0.847 | -0.045 | 0.055 | 0.005 | 0.005 |
| Anxiety | Age | -0.083 | 0.010 | -7.985 | 0.000 | -0.104 | -0.063 | -0.133 | -0.079 |
| Social | Gender | -0.255 | 0.061 | -4.187 | 0.000 | -0.375 | -0.136 | -0.093 | -0.185 |
| Social | Level of education | 0.041 | 0.031 | 1.311 | 0.190 | -0.020 | 0.101 | 0.031 | 0.029 |
| Social | Social grade | -0.018 | 0.033 | -0.546 | 0.585 | -0.082 | 0.046 | -0.014 | -0.013 |
| Social | Age | -0.139 | 0.019 | -7.349 | 0.000 | -0.177 | -0.102 | -0.169 | -0.101 |
| **Covariance** |  |  |  |  |  |  |  |  |  |
| Enhancement | Social | 0.807 | 0.069 | 11.679 | 0.000 | 0.672 | 0.942 | 0.418 | 0.418 |
| Conformity | Social | 0.495 | 0.042 | 11.661 | 0.000 | 0.412 | 0.578 | 0.343 | 0.343 |
| Anxiety | Social | 0.483 | 0.042 | 11.392 | 0.000 | 0.399 | 0.566 | 0.344 | 0.344 |
| Depression | Social | 0.209 | 0.026 | 8.127 | 0.000 | 0.158 | 0.259 | 0.193 | 0.193 |
| Conformity | Anxiety | 0.340 | 0.025 | 13.605 | 0.000 | 0.291 | 0.389 | 0.308 | 0.308 |
| Conformity | Depression | 0.144 | 0.015 | 9.381 | 0.000 | 0.114 | 0.174 | 0.169 | 0.169 |
| Depression | Anxiety | 0.242 | 0.018 | 13.731 | 0.000 | 0.207 | 0.276 | 0.293 | 0.293 |
| Enhancement | Depression | 0.243 | 0.030 | 8.077 | 0.000 | 0.184 | 0.302 | 0.214 | 0.214 |
| Enhancement | Anxiety | 0.422 | 0.042 | 10.038 | 0.000 | 0.340 | 0.504 | 0.285 | 0.285 |
| Conformity | Enhancement | 0.232 | 0.035 | 6.593 | 0.000 | 0.163 | 0.301 | 0.153 | 0.153 |
| At least monthly NoLo consumption | t1 | 1.368 | 0.195 | 7.022 | 0.000 | 0.986 | 1.749 | 1.353 | 1.353 |
| At least monthly NoLo consumption | At least monthly NoLo consumption | 0.980 | 0.000 |  |  | 0.980 | 0.980 | 0.960 | 0.960 |
| AUDIT C | AUDIT C | 5.174 | 0.177 | 29.233 | 0.000 | 4.827 | 5.521 | 0.798 | 0.798 |
| Conformity | Conformity | 1.136 | 0.049 | 23.293 | 0.000 | 1.041 | 1.232 | 0.990 | 0.990 |
| Enhancement | Enhancement | 2.038 | 0.121 | 16.868 | 0.000 | 1.801 | 2.275 | 0.988 | 0.988 |
| Depression | Depression | 0.636 | 0.020 | 31.099 | 0.000 | 0.596 | 0.676 | 0.958 | 0.958 |
| Anxiety | Anxiety | 1.072 | 0.044 | 24.201 | 0.000 | 0.985 | 1.158 | 0.977 | 0.977 |
| Social | Social | 1.831 | 0.101 | 18.138 | 0.000 | 1.633 | 2.029 | 0.961 | 0.961 |
| Level of education | Level of education | 1.089 | 0.000 |  |  | 1.089 | 1.089 | 1.000 | 1.089 |
| Level of education | Gender | 0.019 | 0.000 |  |  | 0.019 | 0.019 | 0.036 | 0.019 |
| Level of education | Social grade | 0.460 | 0.000 |  |  | 0.460 | 0.460 | 0.409 | 0.460 |
| Level of education | Age | -0.023 | 0.000 |  |  | -0.023 | -0.023 | -0.013 | -0.023 |
| Gender | Gender | 0.250 | 0.000 |  |  | 0.250 | 0.250 | 1.000 | 0.250 |
| Gender | Social grade | -0.006 | 0.000 |  |  | -0.006 | -0.006 | -0.012 | -0.006 |
| Gender | Age | 0.008 | 0.000 |  |  | 0.008 | 0.008 | 0.009 | 0.008 |
| Social grade | Social grade | 1.163 | 0.000 |  |  | 1.163 | 1.163 | 1.000 | 1.163 |
| Social grade | Age | 0.103 | 0.000 |  |  | 0.103 | 0.103 | 0.057 | 0.103 |
| **Variances** |  |  |  |  |  |  |  |  |  |
| Age | Age | 2.815 | 0.000 |  |  | 2.815 | 2.815 | 1.000 | 2.815 |
| At least monthly NoLo consumption | At least monthly NoLo consumption | 1.000 | 0.000 |  |  | 1.000 | 1.000 | 1.000 | 1.000 |
| At least monthly NoLo consumption |  | 0.000 | 0.000 |  |  | 0.000 | 0.000 | 0.000 | 0.000 |
| AUDIT C |  | 3.521 | 0.326 | 10.801 | 0.000 | 2.882 | 4.159 | 1.383 | 1.383 |
| Conformity |  | 2.016 | 0.122 | 16.550 | 0.000 | 1.778 | 2.255 | 1.882 | 1.882 |
| Enhancement |  | 2.565 | 0.162 | 15.803 | 0.000 | 2.247 | 2.884 | 1.786 | 1.786 |
| Depression |  | 1.664 | 0.100 | 16.567 | 0.000 | 1.468 | 1.861 | 2.043 | 2.043 |
| Anxiety |  | 2.270 | 0.119 | 19.122 | 0.000 | 2.037 | 2.502 | 2.167 | 2.167 |
| Social |  | 3.492 | 0.155 | 22.478 | 0.000 | 3.188 | 3.797 | 2.531 | 2.531 |
| Level of education |  | 2.394 | 0.000 |  |  | 2.394 | 2.394 | 2.294 | 2.394 |
| Gender |  | 1.490 | 0.000 |  |  | 1.490 | 1.490 | 2.981 | 1.490 |
| Social grade |  | 2.711 | 0.000 |  |  | 2.711 | 2.711 | 2.513 | 2.711 |
| Age |  | 3.746 | 0.000 |  |  | 3.746 | 3.746 | 2.233 | 3.746 |
| **Total** |  | **0.193** | **0.093** | **2.074** | **0.038** | **0.011** | **0.375** | **0.301** | **0.258** |

Significant direct pathways are highlighted.

AUDIT, Alcohol Use Disorder Identification Test; CI, confidence interval; NoLo, alcohol-free and low-alcohol.

**Table S2. Model parameter estimates for direct model with outliers removed**

|  |  |  |  |  |  | **95% CI** | |  |  |
| --- | --- | --- | --- | --- | --- | --- | --- | --- | --- |
| **Dependent variable** | **Predictor variable** | **Unstandardised coefficient** | **Standard error** | **z** | **P value** | **lower** | **upper** | **Standardised coefficient** | **Partially standardised coefficient** |
| **Regressions** |  |  |  |  |  |  |  |  |  |
| At least monthly NoLo consumption | Level of education | 0.1075 | 0.0327 | 3.2937 | 0.0010 | 0.0435 | 0.1715 | 0.1107 | 0.1063 |
| At least monthly NoLo consumption | AUDIT C | 0.0482 | 0.0140 | 3.4375 | 0.0006 | 0.0207 | 0.0757 | 0.1188 | 0.1188 |
| At least monthly NoLo consumption | Gender | -0.0712 | 0.0641 | -1.1114 | 0.2664 | -0.1968 | 0.0544 | -0.0352 | -0.0704 |
| At least monthly NoLo consumption | Social | -0.0411 | 0.0264 | -1.5585 | 0.1191 | -0.0928 | 0.0106 | -0.0554 | -0.0554 |
| At least monthly NoLo consumption | Enhancement | 0.0134 | 0.0246 | 0.5444 | 0.5862 | -0.0349 | 0.0617 | 0.0189 | 0.0189 |
| At least monthly NoLo consumption | Depression | 0.0186 | 0.0569 | 0.3261 | 0.7443 | -0.0930 | 0.1301 | 0.0106 | 0.0106 |
| At least monthly NoLo consumption | Conformity | 0.0627 | 0.0308 | 2.0369 | 0.0417 | 0.0024 | 0.1231 | 0.0642 | 0.0642 |
| At least monthly NoLo consumption | Anxiety | 0.0446 | 0.0365 | 1.2221 | 0.2217 | -0.0269 | 0.1162 | 0.0435 | 0.0435 |
| At least monthly NoLo consumption | Social grade | 0.0522 | 0.0351 | 1.4858 | 0.1373 | -0.0167 | 0.1211 | 0.0556 | 0.0516 |
| At least monthly NoLo consumption | Age | -0.0052 | 0.0196 | -0.2665 | 0.7898 | -0.0437 | 0.0332 | -0.0086 | -0.0052 |
| AUDIT C | Gender | -0.8816 | 0.1047 | -8.4193 | 0.0000 | -1.0868 | -0.6763 | -0.1768 | -0.3537 |
| AUDIT C | Social grade | 0.0847 | 0.0540 | 1.5700 | 0.1164 | -0.0211 | 0.1905 | 0.0366 | 0.0340 |
| AUDIT C | Level of education | -0.0903 | 0.0528 | -1.7112 | 0.0870 | -0.1937 | 0.0131 | -0.0377 | -0.0362 |
| AUDIT C | Conformity | -0.0481 | 0.0532 | -0.9034 | 0.3663 | -0.1523 | 0.0562 | -0.0200 | -0.0200 |
| AUDIT C | Enhancement | 0.4017 | 0.0365 | 10.9999 | 0.0000 | 0.3301 | 0.4733 | 0.2300 | 0.2300 |
| AUDIT C | Depression | 0.3939 | 0.0792 | 4.9748 | 0.0000 | 0.2387 | 0.5490 | 0.0909 | 0.0909 |
| AUDIT C | Social | 0.3260 | 0.0413 | 7.9013 | 0.0000 | 0.2452 | 0.4069 | 0.1785 | 0.1785 |
| AUDIT C | Age | -0.0552 | 0.0322 | -1.7123 | 0.0868 | -0.1183 | 0.0080 | -0.0370 | -0.0221 |
| AUDIT C | Anxiety | 0.0677 | 0.0509 | 1.3283 | 0.1841 | -0.0322 | 0.1675 | 0.0268 | 0.0268 |
| Conformity | Gender | -0.1839 | 0.0469 | -3.9171 | 0.0001 | -0.2759 | -0.0919 | -0.0888 | -0.1776 |
| Conformity | Level of education | -0.0159 | 0.0233 | -0.6825 | 0.4949 | -0.0616 | 0.0298 | -0.0160 | -0.0154 |
| Conformity | Social grade | -0.0190 | 0.0246 | -0.7712 | 0.4406 | -0.0673 | 0.0293 | -0.0198 | -0.0183 |
| Conformity | Age | -0.0040 | 0.0138 | -0.2896 | 0.7721 | -0.0310 | 0.0230 | -0.0064 | -0.0038 |
| Enhancement | Gender | -0.1993 | 0.0637 | -3.1274 | 0.0018 | -0.3241 | -0.0744 | -0.0698 | -0.1396 |
| Enhancement | Level of education | 0.1226 | 0.0329 | 3.7220 | 0.0002 | 0.0580 | 0.1872 | 0.0895 | 0.0859 |
| Enhancement | Social grade | 0.0299 | 0.0340 | 0.8805 | 0.3786 | -0.0367 | 0.0965 | 0.0226 | 0.0210 |
| Enhancement | Age | 0.0284 | 0.0197 | 1.4413 | 0.1495 | -0.0102 | 0.0671 | 0.0333 | 0.0199 |
| Depression | Social grade | -0.0453 | 0.0131 | -3.4483 | 0.0006 | -0.0711 | -0.0196 | -0.0848 | -0.0787 |
| Depression | Gender | 0.0249 | 0.0257 | 0.9683 | 0.3329 | -0.0255 | 0.0753 | 0.0216 | 0.0433 |
| Depression | Level of education | 0.0101 | 0.0140 | 0.7198 | 0.4716 | -0.0173 | 0.0375 | 0.0182 | 0.0175 |
| Depression | Age | -0.0502 | 0.0080 | -6.2786 | 0.0000 | -0.0659 | -0.0345 | -0.1458 | -0.0873 |
| Anxiety | Social grade | -0.0517 | 0.0243 | -2.1272 | 0.0334 | -0.0993 | -0.0041 | -0.0564 | -0.0524 |
| Anxiety | Gender | 0.0375 | 0.0472 | 0.7944 | 0.4270 | -0.0550 | 0.1299 | 0.0190 | 0.0380 |
| Anxiety | Level of education | 0.0054 | 0.0242 | 0.2231 | 0.8235 | -0.0421 | 0.0529 | 0.0057 | 0.0055 |
| Anxiety | Age | -0.0502 | 0.0080 | -6.2786 | 0.0000 | -0.0659 | -0.0345 | -0.0851 | -0.0509 |
| Social | Gender | -0.2657 | 0.0609 | -4.3634 | 0.0000 | -0.3850 | -0.1463 | -0.0973 | -0.1947 |
| Social | Level of education | 0.0455 | 0.0309 | 1.4742 | 0.1404 | -0.0150 | 0.1060 | 0.0347 | 0.0334 |
| Social | Social grade | -0.0122 | 0.0325 | -0.3746 | 0.7080 | -0.0760 | 0.0516 | -0.0096 | -0.0089 |
| Social | Age | -0.1347 | 0.0189 | -7.1106 | 0.0000 | -0.1718 | -0.0976 | -0.1650 | -0.0987 |
| **Covariances** |  |  |  |  |  |  |  |  |  |
| Enhancement | Social | 0.7911 | 0.0676 | 11.7019 | 0.0000 | 0.6586 | 0.9236 | 0.4176 | 0.4176 |
| Conformity | Social | 0.4513 | 0.0391 | 11.5394 | 0.0000 | 0.3746 | 0.5280 | 0.3274 | 0.3274 |
| Anxiety | Social | 0.4170 | 0.0380 | 10.9640 | 0.0000 | 0.3424 | 0.4915 | 0.3176 | 0.3176 |
| Depression | Social | 0.1307 | 0.0189 | 6.9251 | 0.0000 | 0.0937 | 0.1677 | 0.1723 | 0.1723 |
| Conformity | Anxiety | 0.2741 | 0.0213 | 12.8924 | 0.0000 | 0.2324 | 0.3157 | 0.2710 | 0.2710 |
| Conformity | Depression | 0.0697 | 0.0106 | 6.5563 | 0.0000 | 0.0489 | 0.0905 | 0.1193 | 0.1193 |
| Depression | Anxiety | 0.1328 | 0.0105 | 12.5970 | 0.0000 | 0.1122 | 0.1535 | 0.2388 | 0.2388 |
| Enhancement | Depression | 0.1367 | 0.0203 | 6.7300 | 0.0000 | 0.0969 | 0.1765 | 0.1703 | 0.1703 |
| Enhancement | Anxiety | 0.3859 | 0.0396 | 9.7505 | 0.0000 | 0.3083 | 0.4635 | 0.2778 | 0.2778 |
| Conformity | Enhancement | 0.1995 | 0.0332 | 6.0065 | 0.0000 | 0.1344 | 0.2645 | 0.1367 | 0.1367 |
| At least monthly NoLo consumption | t1 | 1.4280 | 0.2028 | 7.0405 | 0.0000 | 1.0305 | 1.8255 | 1.4113 | 1.4113 |
| At least monthly NoLo consumption | At least monthly NoLo consumption | 0.9777 | 0.0000 |  |  | 0.9777 | 0.9777 | 0.9550 | 0.9550 |
| AUDIT C | AUDIT C | 5.0304 | 0.1770 | 28.4151 | 0.0000 | 4.6835 | 5.3774 | 0.8098 | 0.8098 |
| Conformity | Conformity | 1.0619 | 0.0444 | 23.9109 | 0.0000 | 0.9749 | 1.1490 | 0.9911 | 0.9911 |
| Enhancement | Enhancement | 2.0049 | 0.1188 | 16.8692 | 0.0000 | 1.7719 | 2.2378 | 0.9845 | 0.9845 |
| Depression | Depression | 0.3213 | 0.0088 | 36.4529 | 0.0000 | 0.3041 | 0.3386 | 0.9706 | 0.9706 |
| Anxiety | Anxiety | 0.9628 | 0.0382 | 25.1747 | 0.0000 | 0.8878 | 1.0378 | 0.9890 | 0.9890 |
| Social | Social | 1.7897 | 0.0983 | 18.2066 | 0.0000 | 1.5970 | 1.9824 | 0.9614 | 0.9614 |
| Level of education | Level of education | 1.0846 | 0.0000 |  |  | 1.0846 | 1.0846 | 1.0000 | 1.0846 |
| Level of education | Gender | 0.0187 | 0.0000 |  |  | 0.0187 | 0.0187 | 0.0359 | 0.0187 |
| Level of education | Social grade | 0.4508 | 0.0000 |  |  | 0.4508 | 0.4508 | 0.4017 | 0.4508 |
| Level of education | Age | -0.0417 | 0.0000 |  |  | -0.0417 | -0.0417 | -0.0240 | -0.0417 |
| Gender | Gender | 0.2499 | 0.0000 |  |  | 0.2499 | 0.2499 | 1.0000 | 0.2499 |
| Gender | Social grade | -0.0067 | 0.0000 |  |  | -0.0067 | -0.0067 | -0.0125 | -0.0067 |
| Gender | Age | 0.0182 | 0.0000 |  |  | 0.0182 | 0.0182 | 0.0217 | 0.0182 |
| Social grade | Social grade | 1.1611 | 0.0000 |  |  | 1.1611 | 1.1611 | 1.0000 | 1.1611 |
| Social grade | Age | 0.0944 | 0.0000 |  |  | 0.0944 | 0.0944 | 0.0524 | 0.0944 |
| **Variances** |  |  |  |  |  |  |  |  |  |
| Age | Age | 2.7936 | 0.0000 |  |  | 2.7936 | 2.7936 | 1.0000 | 2.7936 |
| At least monthly NoLo consumption | At least monthly NoLo consumption | 1.0000 | 0.0000 |  |  | 1.0000 | 1.0000 | 1.0000 | 1.0000 |
| At least monthly NoLo consumption |  | 0.0000 | 0.0000 |  |  | 0.0000 | 0.0000 | 0.0000 | 0.0000 |
| AUDIT C |  | 3.3850 | 0.3284 | 10.3072 | 0.0000 | 2.7414 | 4.0287 | 1.3581 | 1.3581 |
| Conformity |  | 1.9562 | 0.1200 | 16.2987 | 0.0000 | 1.7210 | 2.1915 | 1.8899 | 1.8899 |
| Enhancement |  | 2.4970 | 0.1639 | 15.2340 | 0.0000 | 2.1758 | 2.8183 | 1.7498 | 1.7498 |
| Depression |  | 1.3485 | 0.0660 | 20.4354 | 0.0000 | 1.2192 | 1.4779 | 2.3437 | 2.3437 |
| Anxiety |  | 2.1542 | 0.1165 | 18.4984 | 0.0000 | 1.9260 | 2.3825 | 2.1833 | 2.1833 |
| Social |  | 3.4364 | 0.1561 | 22.0185 | 0.0000 | 3.1305 | 3.7423 | 2.5186 | 2.5186 |
| Level of education |  | 2.4054 | 0.0000 |  |  | 2.4054 | 2.4054 | 2.3097 | 2.4054 |
| Gender |  | 1.4903 | 0.0000 |  |  | 1.4903 | 1.4903 | 2.9811 | 1.4903 |
| Social grade |  | 2.7236 | 0.0000 |  |  | 2.7236 | 2.7236 | 2.5276 | 2.7236 |
| Age |  | 3.7649 | 0.0000 |  |  | 3.7649 | 3.7649 | 2.2525 | 3.7649 |
| **total** |  | 0.2298 | 0.1015 | 2.2627 | 0.0237 | 0.0307 | 0.4288 | 0.3230 | 0.2829 |

Significant direct pathways are highlighted.

AUDIT, Alcohol Use Disorder Identification Test; CI, confidence interval; NoLo, alcohol-free and low-alcohol.

**Table S3. Fully mediated model (bootstrapped)**

|  |  |  |  |  |  |  | **95% CI** | |  | **Bootstrapped 95% CI** | |
| --- | --- | --- | --- | --- | --- | --- | --- | --- | --- | --- | --- |
| **Dependent variable** | **Predictor variable** | **label** | **Unstandardised coefficient** | **SE** | **z** | **P value** | **lower** | **upper** | **Bootstrapped SE** | **lower** | **upper** |
| **Direct pathways** |  |  |  |  |  |  |  |  |  |  |  |
| At least monthly NoLo consumption | Level of education | c2 | 0.106 | 0.032 | 3.309 | 0.001 | 0.043 | 0.168 | 0.033 | 0.050 | 0.177 |
| At least monthly NoLo consumption | AUDIT C | d | 0.046 | 0.013 | 3.510 | 0.000 | 0.020 | 0.072 | 0.013 | 0.026 | 0.076 |
| At least monthly NoLo consumption | Conformity | e2 | 0.053 | 0.028 | 1.914 | 0.056 | -0.001 | 0.108 | 0.028 | 0.009 | 0.119 |
| Conformity | Gender | a3 | -0.210 | 0.048 | -4.335 | 0.000 | -0.304 | -0.115 | 0.045 | -0.292 | -0.117 |
| Enhancement | Gender | a5 | -0.184 | 0.064 | -2.901 | 0.004 | -0.309 | -0.060 | 0.060 | -0.244 | -0.009 |
| Enhancement | Level of education | c5 | 0.107 | 0.033 | 3.258 | 0.001 | 0.043 | 0.171 | 0.032 | 0.046 | 0.170 |
| Depression | Social grade | b6 | -0.062 | 0.021 | -2.967 | 0.003 | -0.104 | -0.021 | 0.018 | -0.082 | -0.015 |
| Depression | Age | k6 | -0.046 | 0.012 | -3.921 | 0.000 | -0.069 | -0.023 | 0.012 | -0.066 | -0.019 |
| Anxiety | Social grade | b7 | -0.065 | 0.025 | -2.564 | 0.010 | -0.115 | -0.015 | 0.025 | -0.132 | -0.033 |
| Anxiety | Age | k7 | -0.144 | 0.015 | -9.510 | 0.000 | -0.173 | -0.114 | 0.015 | -0.175 | -0.114 |
| Social | Gender | a4 | -0.255 | 0.061 | -4.187 | 0.000 | -0.375 | -0.136 | 0.057 | -0.310 | -0.096 |
| Social | Age | k4 | -0.139 | 0.019 | -7.349 | 0.000 | -0.177 | -0.102 | 0.017 | -0.167 | -0.100 |
| AUDIT C | Gender | a1 | -0.897 | 0.107 | -8.407 | 0.000 | -1.107 | -0.688 | 0.104 | -1.057 | -0.638 |
| AUDIT C | Enhancement | g1 | 0.422 | 0.037 | 11.414 | 0.000 | 0.350 | 0.494 | 0.043 | 0.335 | 0.501 |
| AUDIT C | Depression | h1 | 0.455 | 0.063 | 7.189 | 0.000 | 0.331 | 0.580 | 0.082 | 0.301 | 0.619 |
| AUDIT C | Social | f1 | 0.271 | 0.043 | 6.342 | 0.000 | 0.187 | 0.354 | 0.047 | 0.189 | 0.377 |
| AUDIT C | Age | k1 | -0.070 | 0.033 | -2.111 | 0.035 | -0.135 | -0.005 | 0.032 | -0.153 | -0.028 |
| AUDIT C | Level of education | c1 | -0.094 | 0.055 | -1.715 | 0.086 | -0.202 | 0.013 | 0.055 | -0.163 | 0.047 |
| Enhancement | Social |  | 0.814 | 0.071 | 11.510 | 0.000 | 0.675 | 0.953 | 0.044 | 0.742 | 0.912 |
| Conformity | Social |  | 0.485 | 0.042 | 11.475 | 0.000 | 0.402 | 0.568 | 0.034 | 0.400 | 0.529 |
| Anxiety | Social |  | 0.483 | 0.043 | 11.305 | 0.000 | 0.399 | 0.567 | 0.039 | 0.473 | 0.625 |
| Depression | Social |  | 0.211 | 0.026 | 8.187 | 0.000 | 0.160 | 0.261 | 0.031 | 0.194 | 0.310 |
| Conformity | Anxiety |  | 0.340 | 0.025 | 13.605 | 0.000 | 0.291 | 0.389 | 0.033 | 0.291 | 0.419 |
| Conformity | Depression |  | 0.142 | 0.015 | 9.256 | 0.000 | 0.112 | 0.172 | 0.028 | 0.103 | 0.210 |
| Depression | Anxiety |  | 0.242 | 0.018 | 13.666 | 0.000 | 0.207 | 0.277 | 0.033 | 0.225 | 0.350 |
| Enhancement | Depression |  | 0.245 | 0.030 | 8.096 | 0.000 | 0.186 | 0.304 | 0.031 | 0.213 | 0.337 |
| Enhancement | Anxiety |  | 0.423 | 0.042 | 9.955 | 0.000 | 0.340 | 0.506 | 0.041 | 0.406 | 0.563 |
| Conformity | Enhancement |  | 0.224 | 0.035 | 6.453 | 0.000 | 0.156 | 0.292 | 0.034 | 0.175 | 0.309 |
| At least monthly NoLo consumption | t1 |  | 1.365 | 0.177 | 7.714 | 0.000 | 1.018 | 1.712 | 0.154 | 1.226 | 1.807 |
| At least monthly NoLo consumption | At least monthly NoLo consumption |  | 0.982 | 0.000 |  |  | 0.982 | 0.982 | 0.010 | 0.954 | 0.993 |
| Conformity | Conformity |  | 1.137 | 0.049 | 23.283 | 0.000 | 1.041 | 1.232 | 0.049 | 1.008 | 1.200 |
| Enhancement | Enhancement |  | 2.035 | 0.121 | 16.803 | 0.000 | 1.798 | 2.272 | 0.036 | 1.927 | 2.067 |
| Depression | Depression |  | 0.637 | 0.021 | 31.050 | 0.000 | 0.596 | 0.677 | 0.056 | 0.621 | 0.838 |
| Anxiety | Anxiety |  | 1.072 | 0.044 | 24.201 | 0.000 | 0.985 | 1.158 | 0.053 | 1.092 | 1.297 |
| Social | Social |  | 1.841 | 0.103 | 17.896 | 0.000 | 1.640 | 2.043 | 0.037 | 1.763 | 1.910 |
| AUDIT C | AUDIT C |  | 5.191 | 0.182 | 28.591 | 0.000 | 4.836 | 5.547 | 0.156 | 4.903 | 5.501 |
| At least monthly NoLo consumption | Anxiety |  | 0.041 | 0.030 | 1.373 | 0.170 | -0.018 | 0.099 | 0.032 | -0.041 | 0.084 |
| Level of education | Level of education |  | 1.089 | 0.000 |  |  | 1.089 | 1.089 | 0.021 | 1.031 | 1.109 |
| Level of education | Gender |  | 0.019 | 0.000 |  |  | 0.019 | 0.019 | 0.011 | -0.001 | 0.041 |
| Level of education | Social grade |  | 0.460 | 0.000 |  |  | 0.460 | 0.460 | 0.025 | 0.450 | 0.545 |
| Level of education | Age |  | -0.023 | 0.000 |  |  | -0.023 | -0.023 | 0.038 | -0.081 | 0.066 |
| Gender | Gender |  | 0.250 | 0.000 |  |  | 0.250 | 0.250 | 0.000 | 0.249 | 0.250 |
| Gender | Social grade |  | -0.006 | 0.000 |  |  | -0.006 | -0.006 | 0.013 | -0.031 | 0.020 |
| Gender | Age |  | 0.008 | 0.000 |  |  | 0.008 | 0.008 | 0.019 | -0.003 | 0.069 |
| Social grade | Social grade |  | 1.163 | 0.000 |  |  | 1.163 | 1.163 | 0.022 | 1.265 | 1.351 |
| Social grade | Age |  | 0.103 | 0.000 |  |  | 0.103 | 0.103 | 0.045 | 0.120 | 0.296 |
| Age | Age |  | 2.815 | 0.000 |  |  | 2.815 | 2.815 | 0.053 | 2.779 | 2.987 |
| At least monthly NoLo consumption | At least monthly NoLo consumption |  | 1.000 | 0.000 |  |  | 1.000 | 1.000 | 0.000 | 1.000 | 1.000 |
| At least monthly NoLo consumption |  |  | 0.000 | 0.000 |  |  | 0.000 | 0.000 | 0.000 | 0.000 | 0.000 |
| Conformity |  |  | 2.016 | 0.122 | 16.550 | 0.000 | 1.778 | 2.255 | 0.108 | 1.733 | 2.153 |
| Enhancement |  |  | 2.565 | 0.162 | 15.803 | 0.000 | 2.247 | 2.884 | 0.137 | 2.198 | 2.751 |
| Depression |  |  | 1.664 | 0.100 | 16.567 | 0.000 | 1.468 | 1.861 | 0.094 | 1.474 | 1.834 |
| Anxiety |  |  | 2.270 | 0.119 | 19.122 | 0.000 | 2.037 | 2.502 | 0.122 | 2.023 | 2.488 |
| Social |  |  | 3.492 | 0.155 | 22.478 | 0.000 | 3.188 | 3.797 | 0.137 | 3.073 | 3.623 |
| AUDIT C |  |  | 3.539 | 0.325 | 10.879 | 0.000 | 2.902 | 4.177 | 0.301 | 2.850 | 4.075 |
| Level of education |  |  | 2.394 | 0.000 |  |  | 2.394 | 2.394 | 0.021 | 2.264 | 2.348 |
| Gender |  |  | 1.490 | 0.000 |  |  | 1.490 | 1.490 | 0.010 | 1.481 | 1.522 |
| Social grade |  |  | 2.711 | 0.000 |  |  | 2.711 | 2.711 | 0.026 | 2.420 | 2.523 |
| Age |  |  | 3.746 | 0.000 |  |  | 3.746 | 3.746 | 0.038 | 3.384 | 3.532 |
| **indirect pathways** |  |  |  |  |  |  |  |  |  |  |  |
| indirect1 | a3*e2 | indirect1 | -0.011 | 0.006 | -1.759 | 0.079 | -0.024 | 0.001 | 0.006 | -0.026 | -0.002 |
| indirect5 | a1*d | indirect5 | -0.041 | 0.013 | -3.221 | 0.001 | -0.066 | -0.016 | 0.012 | -0.068 | -0.020 |
| indirect6 | a4*f1*d | indirect6 | -0.003 | 0.001 | -2.476 | 0.013 | -0.006 | -0.001 | 0.001 | -0.006 | -0.001 |
| indirect7 | k4*f1*d | indirect7 | -0.002 | 0.001 | -2.744 | 0.006 | -0.003 | 0.000 | 0.001 | -0.003 | -0.001 |
| indirect14a | b6*h1*d | indirect14a | -0.001 | 0.001 | -2.170 | 0.030 | -0.002 | 0.000 | 0.001 | -0.002 | 0.000 |
| indirect17 | a5*g1*d | indirect17 | -0.004 | 0.002 | -2.173 | 0.030 | -0.007 | 0.000 | 0.002 | -0.006 | 0.000 |
| indirect19 | c5*g1*d | indirect19 | 0.002 | 0.001 | 2.301 | 0.021 | 0.000 | 0.004 | 0.001 | 0.001 | 0.004 |
| indirect20 | c1*d | indirect20 | -0.0043406 | 0.00282534 | -1.53629315 | 0.1244665 | -0.0098781 | 0.00119701 | 0.0029568 | -0.009344 | 0.00257319 |
| indirect26 | k6*h1*d | indirect26 | -0.001 | 0.000 | -2.425 | 0.015 | -0.002 | 0.000 | 0.000 | -0.002 | 0.000 |
| indirect28 | k1*d | indirect28 | -0.003 | 0.002 | -1.792 | 0.073 | -0.007 | 0.000 | 0.002 | -0.009 | -0.001 |
| overallindirect | indirect1+indirect5+indirect6+indirect7+indirect14a+indirect17+indirect19+indirect20+indirect26+indirect28 | overallindirect | -0.069 | 0.019 | -3.682 | 0.000 | -0.105 | -0.032 | 0.018 | -0.106 | -0.037 |
| total | c2+d+e2+overallindirect | total | 0.136 | 0.040 | 3.441 | 0.001 | 0.059 | 0.214 | 0.041 | 0.080 | 0.238 |

Significant direct and indirect pathways highlighted.

AUDIT, Alcohol Use Disorder Identification Test; CI, confidence interval; NoLo, alcohol-free and low-alcohol.

**Table S4. Selected Mediated Model**

|  |  |  |  |  |  |  | **95% CI** | |  | **Bootstrapped 95% CI** | |
| --- | --- | --- | --- | --- | --- | --- | --- | --- | --- | --- | --- |
| **Dependent variable** | **Predictor variable** | **Label** | **Unstandardised coefficient** | **SE** | **z** | **P value** | **lower** | **upper** | **Bootstrapped SE** | **lower** | **upper** |
| **Direct pathways** |  |  |  |  |  |  |  |  |  |  |  |
| At least monthly NoLo consumption | Level of education | c2 | 0.102 | 0.032 | 3.185 | 0.001 | 0.039 | 0.164 | 0.033 | 0.047 | 0.174 |
| At least monthly NoLo consumption | AUDIT C | d | 0.046 | 0.013 | 3.507 | 0.000 | 0.020 | 0.072 | 0.013 | 0.026 | 0.076 |
| At least monthly NoLo consumption | Conformity | e2 | 0.053 | 0.028 | 1.915 | 0.055 | -0.001 | 0.108 | 0.028 | 0.010 | 0.119 |
| Conformity | Gender | a3 | -0.210 | 0.048 | -4.335 | 0.000 | -0.304 | -0.115 | 0.045 | -0.292 | -0.117 |
| Enhancement | Gender | a5 | -0.184 | 0.064 | -2.901 | 0.004 | -0.309 | -0.060 | 0.060 | -0.244 | -0.009 |
| Enhancement | Level of education | c5 | 0.095 | 0.034 | 2.791 | 0.005 | 0.028 | 0.162 | 0.033 | 0.036 | 0.167 |
| Depression | Social grade | b6 | -0.062 | 0.021 | -2.965 | 0.003 | -0.104 | -0.021 | 0.018 | -0.082 | -0.015 |
| Depression | Age | k6 | -0.046 | 0.012 | -3.921 | 0.000 | -0.069 | -0.023 | 0.012 | -0.066 | -0.019 |
| Social | Gender | a4 | -0.255 | 0.061 | -4.187 | 0.000 | -0.375 | -0.136 | 0.057 | -0.310 | -0.096 |
| Social | Age | k4 | -0.139 | 0.019 | -7.349 | 0.000 | -0.177 | -0.102 | 0.017 | -0.167 | -0.100 |
| AUDIT C | Gender | a1 | -0.898 | 0.107 | -8.416 | 0.000 | -1.107 | -0.689 | 0.104 | -1.056 | -0.642 |
| AUDIT C | Enhancement | g1 | 0.408 | 0.037 | 11.089 | 0.000 | 0.336 | 0.480 | 0.041 | 0.325 | 0.482 |
| AUDIT C | Depression | h1 | 0.458 | 0.063 | 7.298 | 0.000 | 0.335 | 0.581 | 0.082 | 0.298 | 0.620 |
| AUDIT C | Social | f1 | 0.278 | 0.042 | 6.616 | 0.000 | 0.195 | 0.360 | 0.046 | 0.196 | 0.372 |
| AUDIT C | Age | k1 | -0.069 | 0.033 | -2.079 | 0.038 | -0.134 | -0.004 | 0.031 | -0.153 | -0.029 |
| Enhancement | Social |  | 0.815 | 0.070 | 11.596 | 0.000 | 0.677 | 0.953 | 0.044 | 0.747 | 0.918 |
| Conformity | Social |  | 0.485 | 0.042 | 11.475 | 0.000 | 0.402 | 0.568 | 0.034 | 0.400 | 0.529 |
| Depression | Social |  | 0.211 | 0.026 | 8.188 | 0.000 | 0.160 | 0.261 | 0.031 | 0.195 | 0.311 |
| Conformity | Depression |  | 0.142 | 0.015 | 9.253 | 0.000 | 0.112 | 0.172 | 0.028 | 0.104 | 0.210 |
| Enhancement | Depression |  | 0.246 | 0.030 | 8.134 | 0.000 | 0.187 | 0.305 | 0.031 | 0.216 | 0.339 |
| Conformity | Enhancement |  | 0.224 | 0.035 | 6.459 | 0.000 | 0.156 | 0.292 | 0.035 | 0.176 | 0.311 |
| At least monthly NoLo consumption | t1 |  | 1.365 | 0.177 | 7.716 | 0.000 | 1.019 | 1.712 | 0.154 | 1.229 | 1.805 |
| At least monthly NoLo consumption | At least monthly NoLo consumption |  | 0.982 | 0.000 |  |  | 0.982 | 0.982 | 0.010 | 0.953 | 0.993 |
| Conformity | Conformity |  | 1.137 | 0.049 | 23.283 | 0.000 | 1.041 | 1.232 | 0.049 | 1.008 | 1.200 |
| Enhancement | Enhancement |  | 2.045 | 0.121 | 16.876 | 0.000 | 1.808 | 2.283 | 0.038 | 1.940 | 2.082 |
| Depression | Depression |  | 0.637 | 0.020 | 31.096 | 0.000 | 0.597 | 0.677 | 0.056 | 0.622 | 0.839 |
| Social | Social |  | 1.841 | 0.103 | 17.932 | 0.000 | 1.640 | 2.042 | 0.038 | 1.766 | 1.915 |
| AUDIT C | AUDIT C |  | 5.206 | 0.181 | 28.811 | 0.000 | 4.852 | 5.560 | 0.151 | 4.934 | 5.530 |
| Level of education | Level of education |  | 1.089 | 0.000 |  |  | 1.089 | 1.089 | 0.021 | 1.031 | 1.109 |
| Level of education | Gender |  | 0.019 | 0.000 |  |  | 0.019 | 0.019 | 0.011 | -0.001 | 0.041 |
| Level of education | Social grade |  | 0.460 | 0.000 |  |  | 0.460 | 0.460 | 0.025 | 0.450 | 0.545 |
| Level of education | Age |  | -0.023 | 0.000 |  |  | -0.023 | -0.023 | 0.038 | -0.081 | 0.066 |
| Gender | Gender |  | 0.250 | 0.000 |  |  | 0.250 | 0.250 | 0.000 | 0.249 | 0.250 |
| Gender | Social grade |  | -0.006 | 0.000 |  |  | -0.006 | -0.006 | 0.013 | -0.031 | 0.020 |
| Gender | Age |  | 0.008 | 0.000 |  |  | 0.008 | 0.008 | 0.019 | -0.003 | 0.069 |
| Social grade | Social grade |  | 1.163 | 0.000 |  |  | 1.163 | 1.163 | 0.022 | 1.265 | 1.351 |
| Social grade | Age |  | 0.103 | 0.000 |  |  | 0.103 | 0.103 | 0.045 | 0.120 | 0.296 |
| Age | Age |  | 2.815 | 0.000 |  |  | 2.815 | 2.815 | 0.053 | 2.779 | 2.987 |
| At least monthly NoLo consumption | At least monthly NoLo consumption |  | 1.000 | 0.000 |  |  | 1.000 | 1.000 | 0.000 | 1.000 | 1.000 |
| At least monthly NoLo consumption |  |  | 0.000 | 0.000 |  |  | 0.000 | 0.000 | 0.000 | 0.000 | 0.000 |
| Conformity |  |  | 2.016 | 0.122 | 16.550 | 0.000 | 1.778 | 2.255 | 0.108 | 1.733 | 2.153 |
| Enhancement |  |  | 2.565 | 0.162 | 15.803 | 0.000 | 2.247 | 2.884 | 0.137 | 2.198 | 2.751 |
| Depression |  |  | 1.664 | 0.100 | 16.567 | 0.000 | 1.468 | 1.861 | 0.094 | 1.474 | 1.834 |
| Social |  |  | 3.492 | 0.155 | 22.478 | 0.000 | 3.188 | 3.797 | 0.137 | 3.073 | 3.623 |
| AUDIT C |  |  | 3.546 | 0.320 | 11.081 | 0.000 | 2.919 | 4.173 | 0.293 | 2.904 | 4.098 |
| Level of education |  |  | 2.394 | 0.000 |  |  | 2.394 | 2.394 | 0.021 | 2.264 | 2.348 |
| Gender |  |  | 1.490 | 0.000 |  |  | 1.490 | 1.490 | 0.010 | 1.481 | 1.522 |
| Social grade |  |  | 2.711 | 0.000 |  |  | 2.711 | 2.711 | 0.026 | 2.420 | 2.523 |
| Age |  |  | 3.746 | 0.000 |  |  | 3.746 | 3.746 | 0.038 | 3.384 | 3.532 |
| **Indirect pathways** |  |  |  |  |  |  |  |  |  |  |  |
| indirect1 | a3*e2 | indirect1 | -0.011 | 0.006 | -1.760 | 0.078 | -0.024 | 0.001 | 0.006 | -0.026 | -0.002 |
| indirect5 | a1*d | indirect5 | -0.041 | 0.013 | -3.218 | 0.001 | -0.066 | -0.016 | 0.012 | -0.068 | -0.021 |
| indirect6 | a4*f1*d | indirect6 | -0.003 | 0.001 | -2.497 | 0.013 | -0.006 | -0.001 | 0.001 | -0.006 | -0.001 |
| indirect7 | k4*f1*d | indirect7 | -0.002 | 0.001 | -2.773 | 0.006 | -0.003 | -0.001 | 0.001 | -0.003 | -0.001 |
| indirect14a | b6*h1*d | indirect14a | -0.001 | 0.001 | -2.174 | 0.030 | -0.003 | 0.000 | 0.001 | -0.002 | 0.000 |
| indirect17 | a5*g1*d | indirect17 | -0.003 | 0.002 | -2.172 | 0.030 | -0.007 | 0.000 | 0.001 | -0.006 | 0.000 |
| indirect19 | c5*g1*d | indirect19 | 0.002 | 0.001 | 2.101 | 0.036 | 0.000 | 0.003 | 0.001 | 0.001 | 0.004 |
| indirect26 | k6*h1*d | indirect26 | -0.001 | 0.000 | -2.429 | 0.015 | -0.002 | 0.000 | 0.000 | -0.002 | 0.000 |
| indirect28 | k1*d | indirect28 | -0.003 | 0.002 | -1.770 | 0.077 | -0.007 | 0.000 | 0.002 | -0.009 | -0.001 |
| overallindirect | indirect1+indirect5+indirect6+indirect14a+indirect17+indirect19+indirect7+indirect26+indirect28 | overallindirect | -0.065 | 0.017 | -3.707 | 0.000 | -0.099 | -0.030 | 0.017 | -0.101 | -0.036 |
| total | c2+d+e2+overallindirect | total | 0.136 | 0.040 | 3.441 | 0.001 | 0.059 | 0.214 | 0.041 | 0.081 | 0.238 |

Significant direct and indirect pathways highlighted.

AUDIT, Alcohol Use Disorder Identification Test; CI, confidence interval; NoLo, alcohol-free and low-alcohol.

**Table S5. Mediated model with outliers removed**

|  |  |  |  |  |  |  | **95% CI** | |  | **Bootstrapped 95% CI** | |
| --- | --- | --- | --- | --- | --- | --- | --- | --- | --- | --- | --- |
| **Dependent variable** | **Predictor variable** | **Label** | **Unstandardised coefficient** | **SE** | **z** | **P value** | **lower** | **upper** | **Bootstrapped SE** | **lower** | **upper** |
| **Direct pathways** |  |  |  |  |  |  |  |  |  |  |  |
| At least monthly NoLo consumption | Level of education | c2 | 0.102 | 0.032 | 3.185 | 0.001 | 0.039 | 0.164 | 0.033 | 0.051 | 0.182 |
| At least monthly NoLo consumption | AUDIT C | d | 0.046 | 0.013 | 3.507 | 0.000 | 0.020 | 0.072 | 0.013 | 0.029 | 0.078 |
| At least monthly NoLo consumption | Conformity | e2 | 0.053 | 0.028 | 1.915 | 0.055 | -0.001 | 0.108 | 0.029 | 0.015 | 0.130 |
| Conformity | Gender | a3 | -0.210 | 0.048 | -4.335 | 0.000 | -0.304 | -0.115 | 0.044 | -0.256 | -0.083 |
| Enhancement | Gender | a5 | -0.184 | 0.064 | -2.901 | 0.004 | -0.309 | -0.060 | 0.064 | -0.269 | -0.020 |
| Enhancement | Level of education | c5 | 0.095 | 0.034 | 2.791 | 0.005 | 0.028 | 0.162 | 0.034 | 0.063 | 0.195 |
| Depression | Social grade | b6 | -0.062 | 0.021 | -2.965 | 0.003 | -0.104 | -0.021 | 0.013 | -0.067 | -0.018 |
| Depression | Age | k6 | -0.046 | 0.012 | -3.921 | 0.000 | -0.069 | -0.023 | 0.007 | -0.027 | 0.002 |
| Social | Gender | a4 | -0.255 | 0.061 | -4.187 | 0.000 | -0.375 | -0.136 | 0.061 | -0.348 | -0.106 |
| Social | Age | k4 | -0.139 | 0.019 | -7.349 | 0.000 | -0.177 | -0.102 | 0.018 | -0.163 | -0.096 |
| AUDIT C | Gender | a1 | -0.898 | 0.107 | -8.416 | 0.000 | -1.107 | -0.689 | 0.103 | -1.007 | -0.610 |
| AUDIT C | Enhancement | g1 | 0.408 | 0.037 | 11.089 | 0.000 | 0.336 | 0.480 | 0.040 | 0.332 | 0.489 |
| AUDIT C | Depression | h1 | 0.458 | 0.063 | 7.298 | 0.000 | 0.335 | 0.581 | 0.116 | 0.180 | 0.617 |
| AUDIT C | Social | f1 | 0.278 | 0.042 | 6.616 | 0.000 | 0.195 | 0.360 | 0.045 | 0.239 | 0.411 |
| AUDIT C | Age | k1 | -0.069 | 0.033 | -2.079 | 0.038 | -0.134 | -0.004 | 0.032 | -0.152 | -0.023 |
| Enhancement | Social |  | 0.815 | 0.070 | 11.596 | 0.000 | 0.677 | 0.953 | 0.042 | 0.730 | 0.894 |
| Conformity | Social |  | 0.485 | 0.042 | 11.475 | 0.000 | 0.402 | 0.568 | 0.033 | 0.361 | 0.492 |
| Depression | Social |  | 0.211 | 0.026 | 8.188 | 0.000 | 0.160 | 0.261 | 0.017 | 0.119 | 0.186 |
| Conformity | Depression |  | 0.142 | 0.015 | 9.253 | 0.000 | 0.112 | 0.172 | 0.016 | 0.050 | 0.111 |
| Enhancement | Depression |  | 0.246 | 0.030 | 8.134 | 0.000 | 0.187 | 0.305 | 0.018 | 0.111 | 0.179 |
| Conformity | Enhancement |  | 0.224 | 0.035 | 6.459 | 0.000 | 0.156 | 0.292 | 0.034 | 0.154 | 0.286 |
| At least monthly NoLo consumption | t1 |  | 1.365 | 0.177 | 7.716 | 0.000 | 1.019 | 1.712 | 0.153 | 1.269 | 1.880 |
| At least monthly NoLo consumption | At least monthly NoLo consumption |  | 0.982 | 0.000 |  |  | 0.982 | 0.982 | 0.010 | 0.951 | 0.990 |
| Conformity | Conformity |  | 1.137 | 0.049 | 23.283 | 0.000 | 1.041 | 1.232 | 0.049 | 0.934 | 1.125 |
| Enhancement | Enhancement |  | 2.045 | 0.121 | 16.876 | 0.000 | 1.808 | 2.283 | 0.037 | 1.903 | 2.045 |
| Depression | Depression |  | 0.637 | 0.020 | 31.096 | 0.000 | 0.597 | 0.677 | 0.025 | 0.278 | 0.373 |
| Social | Social |  | 1.841 | 0.103 | 17.932 | 0.000 | 1.640 | 2.042 | 0.038 | 1.710 | 1.864 |
| AUDIT C | AUDIT C |  | 5.206 | 0.181 | 28.811 | 0.000 | 4.852 | 5.560 | 0.161 | 4.760 | 5.384 |
| Level of education | Level of education |  | 1.089 | 0.000 |  |  | 1.089 | 1.089 | 0.021 | 1.028 | 1.112 |
| Level of education | Gender |  | 0.019 | 0.000 |  |  | 0.019 | 0.019 | 0.011 | -0.002 | 0.042 |
| Level of education | Social grade |  | 0.460 | 0.000 |  |  | 0.460 | 0.460 | 0.026 | 0.438 | 0.539 |
| Level of education | Age |  | -0.023 | 0.000 |  |  | -0.023 | -0.023 | 0.036 | -0.091 | 0.049 |
| Gender | Gender |  | 0.250 | 0.000 |  |  | 0.250 | 0.250 | 0.000 | 0.249 | 0.250 |
| Gender | Social grade |  | -0.006 | 0.000 |  |  | -0.006 | -0.006 | 0.013 | -0.030 | 0.021 |
| Gender | Age |  | 0.008 | 0.000 |  |  | 0.008 | 0.008 | 0.019 | 0.007 | 0.082 |
| Social grade | Social grade |  | 1.163 | 0.000 |  |  | 1.163 | 1.163 | 0.023 | 1.271 | 1.361 |
| Social grade | Age |  | 0.103 | 0.000 |  |  | 0.103 | 0.103 | 0.044 | 0.112 | 0.288 |
| Age | Age |  | 2.815 | 0.000 |  |  | 2.815 | 2.815 | 0.054 | 2.754 | 2.971 |
| At least monthly NoLo consumption | At least monthly NoLo consumption |  | 1.000 | 0.000 |  |  | 1.000 | 1.000 | 0.000 | 1.000 | 1.000 |
| At least monthly NoLo consumption |  |  | 0.000 | 0.000 |  |  | 0.000 | 0.000 | 0.000 | 0.000 | 0.000 |
| Conformity |  |  | 2.016 | 0.122 | 16.550 | 0.000 | 1.778 | 2.255 | 0.105 | 1.669 | 2.070 |
| Enhancement |  |  | 2.565 | 0.162 | 15.803 | 0.000 | 2.247 | 2.884 | 0.144 | 2.116 | 2.684 |
| Depression |  |  | 1.664 | 0.100 | 16.567 | 0.000 | 1.468 | 1.861 | 0.059 | 1.209 | 1.438 |
| Social |  |  | 3.492 | 0.155 | 22.478 | 0.000 | 3.188 | 3.797 | 0.143 | 2.996 | 3.559 |
| AUDIT C |  |  | 3.546 | 0.320 | 11.081 | 0.000 | 2.919 | 4.173 | 0.284 | 2.855 | 3.963 |
| Level of education |  |  | 2.394 | 0.000 |  |  | 2.394 | 2.394 | 0.023 | 2.278 | 2.367 |
| Gender |  |  | 1.490 | 0.000 |  |  | 1.490 | 1.490 | 0.011 | 1.481 | 1.522 |
| Social grade |  |  | 2.711 | 0.000 |  |  | 2.711 | 2.711 | 0.025 | 2.433 | 2.534 |
| Age |  |  | 3.746 | 0.000 |  |  | 3.746 | 3.746 | 0.037 | 3.408 | 3.555 |
| **Indirect pathways** |  |  |  |  |  |  |  |  |  |  |  |
| indirect1 | a3*e2 | indirect1 | -0.011 | 0.006 | -1.760 | 0.078 | -0.024 | 0.001 | 0.006 | -0.024 | -0.002 |
| indirect5 | a1*d | indirect5 | -0.041 | 0.013 | -3.218 | 0.001 | -0.066 | -0.016 | 0.012 | -0.069 | -0.021 |
| indirect6 | a4*f1*d | indirect6 | -0.003 | 0.001 | -2.497 | 0.013 | -0.006 | -0.001 | 0.002 | -0.007 | -0.002 |
| indirect7 | k4*f1*d | indirect7 | -0.002 | 0.001 | -2.773 | 0.006 | -0.003 | -0.001 | 0.001 | -0.004 | -0.001 |
| indirect14a | b6*h1*d | indirect14a | -0.001 | 0.001 | -2.174 | 0.030 | -0.003 | 0.000 | 0.000 | -0.002 | 0.000 |
| indirect17 | a5*g1*d | indirect17 | -0.003 | 0.002 | -2.172 | 0.030 | -0.007 | 0.000 | 0.002 | -0.007 | 0.000 |
| indirect19 | c5*g1*d | indirect19 | 0.002 | 0.001 | 2.101 | 0.036 | 0.000 | 0.003 | 0.001 | 0.001 | 0.005 |
| indirect26 | k6*h1*d | indirect26 | -0.001 | 0.000 | -2.429 | 0.015 | -0.002 | 0.000 | 0.000 | -0.001 | 0.000 |
| indirect28 | k1*d | indirect28 | -0.003 | 0.002 | -1.770 | 0.077 | -0.007 | 0.000 | 0.002 | -0.009 | -0.001 |
| overallindirect | indirect1+indirect5+indirect6+indirect14a+indirect17+indirect19+indirect7+indirect26+indirect28 | overallindirect | -0.065 | 0.017 | -3.707 | 0.000 | -0.099 | -0.030 | 0.016 | -0.102 | -0.040 |
| total | c2+d+e2+overallindirect | total | 0.136 | 0.040 | 3.441 | 0.001 | 0.059 | 0.214 | 0.041 | 0.095 | 0.251 |

AUDIT, Alcohol Use Disorder Identification Test; CI, confidence interval; NoLo, alcohol-free and low-alcohol.

**Table S6. The relationship between hazardous drinking and NoLo consumption without drinking motives included in the model**

|  |  |  | **Bootstrapped results** | |
| --- | --- | --- | --- | --- |
|  | **Standardised estimate β** | **Unstandardised estimate *b*** | **SE** | **95% CI** |
| ***Direct pathways to NoLo*** | |  |  |  |
| Education 🡪 NoLo | 0.126 | 0.122 | 0.029 | 0.064, 0.179 |
| Hazardous drinking 🡪 NoLo | 0.121 | 0.048 | 0.012 | 0.024, 0.072 |
| Conformity 🡪 NoLo | 0.071 | 0.067 | 0.027 | 0.015, 0.120 |
| ***Indirect pathways to NoLo*** | |  |  |  |
| 2. Gender^a^ 🡪 Conformity 🡪 NoLo | -0.007 | -0.014 | 0.030 | -0.027, -0.001 |
| 3. Gender^a^ 🡪 Hazardous drinking 🡪 NoLo | -0.025 | -0.050 | 0.014 | -0.078, -0.022 |
| 6. Age 🡪 Hazardous drinking 🡪 NoLo | -0.010 | -0.006 | 0.002 | -0.011, -0.002 |
| **Total indirect pathways** | -0.042 | -0.070 | 0.037 | -0.105, -0.036 |
| **Total direct and indirect pathways** | 0.276 | 0.166 | 0.037 | 0.094, 0.239 |

^a^Male is the reference category.

CI, confidence interval.
